# GSDMD deficiency ameliorates hyperoxia-induced BPD and ROP in neonatal mice

**DOI:** 10.1038/s41598-022-27201-y

**Published:** 2023-01-04

**Authors:** Sarah Sonny, Huijun Yuan, Shaoyi Chen, Matthew R. Duncan, Pingping Chen, Merline Benny, Karen Young, Kevin K. Park, Augusto F. Schmidt, Shu Wu

**Affiliations:** 1 Neonatology and Batchelor Children Research Institute, University of Miami Miller School of Medicine, 1580 NW 10thAve, Miami, FL 33136 USA; 2 Department of Neurological Surgery, The Miami Project to Cure Paralysis, Miami, FL USA

**Keywords:** Molecular biology, Diseases, Pathogenesis

## Abstract

Bronchopulmonary dysplasia (BPD) and retinopathy of prematurity (ROP) are among the most common morbidities affecting extremely premature infants who receive oxygen therapy. Many clinical studies indicate that BPD is associated with advanced ROP. However, the mechanistic link between hyperoxia, BPD, and ROP remains to be explored. Gasdermin D (GSDMD) is a key executor of inflammasome-induced pyroptosis and inflammation. Inhibition of GSDMD has been shown to attenuate hyperoxia-induced BPD and brain injury in neonatal mice. The objective of this study was to further define the mechanistic roles of GSDMD in the pathogenesis of hyperoxia-induced BPD and ROP in mouse models. Here we show that global GSDMD knockout (GSDMD-KO) protects against hyperoxia-induced BPD by reducing macrophage infiltration, improving alveolarization and vascular development, and decreasing cell death. In addition, GSDMD deficiency prevented hyperoxia-induced ROP by reducing vasoobliteration and neovascularization, improving thinning of multiple retinal tissue layers, and decreasing microglial activation. RNA sequencing analyses of lungs and retinas showed that similar genes, including those from inflammatory, cell death, tissue remodeling, and tissue and vascular developmental signaling pathways, were induced by hyperoxia and impacted by GSDMD-KO in both models. These data highlight the importance of GSDMD in the pathogenesis of BPD and ROP and suggest that targeting GSDMD may be beneficial in preventing and treating BPD and ROP in premature infants.

## Introduction

Each year more than 15 million infants are born preterm worldwide^1^. Extremely premature infants born at less than 28 weeks of gestational age are at great risk of having multi-organ injury and developmental abnormalities that predominantly involve the lung, brain, and eye^2–4^. Born with immature lungs, these premature infants suffer respiratory failure soon after birth and require oxygen (O_2_) therapy to survive. However, life-sustaining high concentration O_2_ therapy (hyperoxia) can cause lung inflammation that ultimately leads to bronchopulmonary dysplasia (BPD), characterized by disrupted alveolar and vascular development and reduced lung function^2^. These premature infants also have immature retinas with underdeveloped retinal vasculature. Exposure to hyperoxia can cause initial obliteration of retinal vasculature which leads to retinal hypoxia and over-production of vascular endothelial growth factor (VEGF) in the retinas that subsequently stimulates neovascularization and progression to retinopathy of prematurity (ROP)^5,6^. ROP is a leading cause of severe vision impairment in children worldwide, and effective treatments are lacking. Although hyperoxia is known to play a critical role in the development and progression of both BPD and ROP, the clinical practice of lowering oxygen saturation by avoiding excessive use of oxygen is associated with increased mortality^7^. While intraocular VEGF antagonists have been recently used to treat severe ROP, they were observed to have possible systemic anti-angiogenic effects that are detrimental to the development of other organs, such as the lung and brain, and they do not correct retinal neuronal injury^8^. Many clinical studies indicate that BPD is associated with advanced ROP^6,9^. However, it is currently unknown if the induction of BPD and ROP by hyperoxia is mechanistically linked, although a common mechanism would be an attractive target for developing therapies for both diseases.

Activation of the inflammasome cascade has been linked to BPD and ROP. Inflammasomes are large macromolecular signaling complexes that control the proteolytic activation of two highly proinflammatory IL-1 family cytokines, IL-1β and IL-18^10^. Previous studies have demonstrated that activation of the NLRP3 (NACHT, LRR, and PYD domains-containing protein 3) inflammasome and increased IL-1β:IL1ra ratio are predictive of the development of BPD^11^. Increased IL-1β and IL-18 were associated with ROP in preterm infants^12^. There is increasing interest in gasdermin D (GSDMD), a 53-kilodalton (kDa) cytosolic protein, which is also a substrate for the inflammasome cascade and was recently found to be a key executor of pyroptosis, a form of programmed inflammatory cell death^13,14^. Activation of the inflammasome pathway by pathogens or host-derived danger signals leads to the activation of inflammatory caspases. Cleavage of GSDMD by these caspases releases a 30-kDa N-terminal domain (p30) that oligomerizes in the cell membrane to form pores, which cause localized cellular swelling, membrane rupture, and cell death, known as pyroptosis. In addition, the pores formed by GSDMD-p30 oligomerization also allow rapid release of active IL-1β and IL-18, resulting in secondary inflammation. Many studies have demonstrated a critical role for GSDMD in regulating pyroptosis and inflammation in various adult diseases. However, there are no reports on the role of GSDMD in neonatal lung and retinal injury in preterm infants, but recent studies from our laboratory have highlighted a critical role for GSDMD in hyperoxia-indued and mechanical ventilation-associated neonatal lung and brain injury in rodent models^15,16^. We showed that GSDMD is activated by hyperoxia in the lungs and brains of neonatal mice and that treatment with a pharmacological inhibitor of GSDMD attenuated hyperoxia-induced BPD-like pathology and brain injury^15^.

In this study, we utilized global GSDMD knockout (GSDMD-KO) mice^13^ and their wildtype (WT) littermates to test the hypothesis that GSDMD acts as a novel mediator of hyperoxia-induced BPD and ROP by inducing cell death and inflammation. We found that hyperoxia-exposed WT mice developed the pathological hallmarks of BPD and ROP. In contrast, these BPD and ROP phenotypes were significantly reduced in hyperoxia-exposed GSDMD-KO mice. We also performed RNA sequencing (RNA-seq) analyses of the lungs and the retinas and found that GSDMD-KO attenuated the effects of hyperoxia on inflammatory, cell death, tissue remodeling, and tissue and vascular developmental signaling pathways. Taken together, our results show that GSDMD deficiency ameliorates hyperoxia-induced BPD and ROP in mouse models and reveal novel GSDMD-regulated gene pathways that are critical in the development and progression of BPD and ROP. Our findings not only fill a gap in understanding the critical role of GSDMD in the pathogenesis of BPD and ROP but also identify potential novel targets for preventing and treating BPD and ROP in premature infants.

## Results

### GSDMD deficiency reduces lung inflammation in hyperoxia-exposed lungs

**Figure 1:**
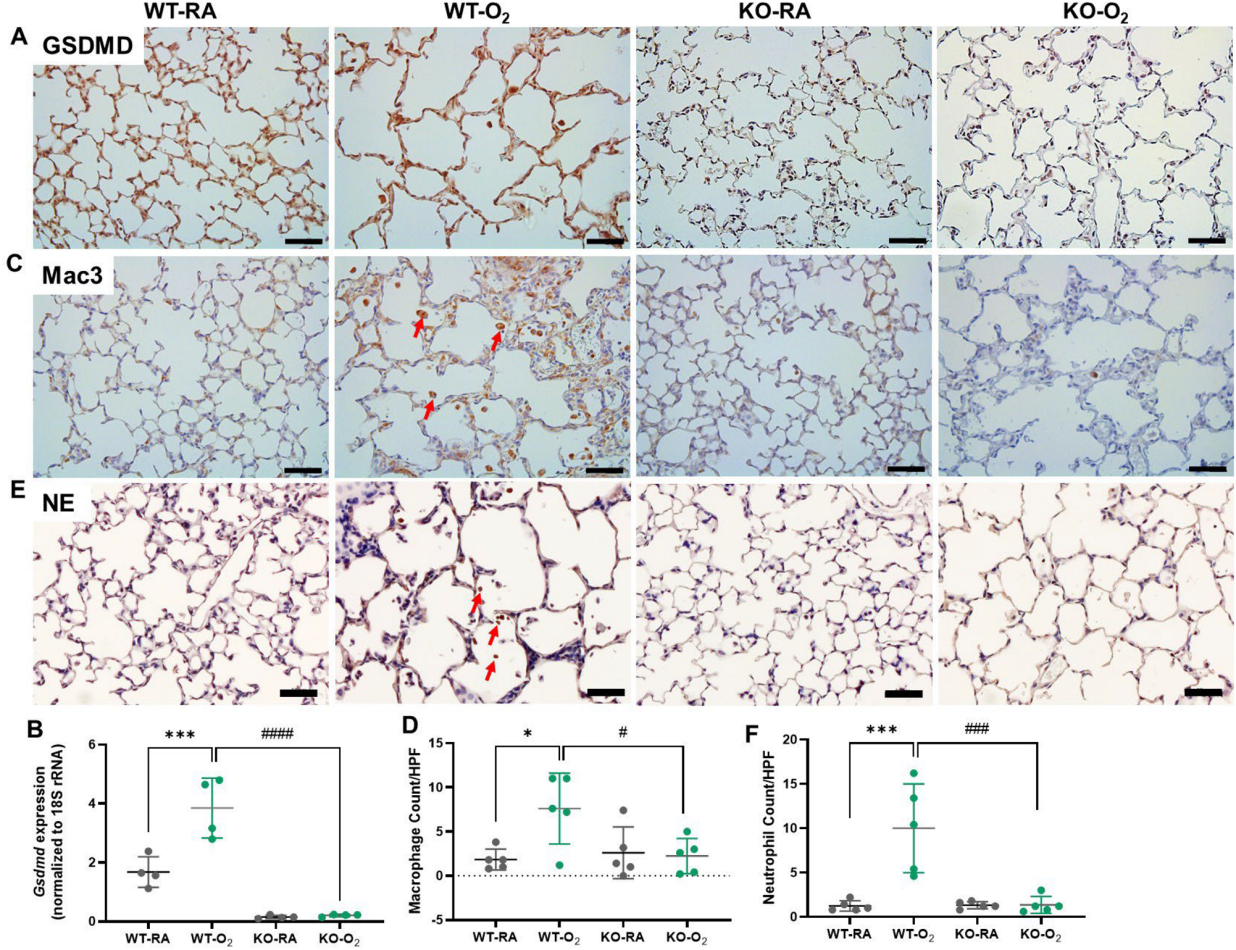
GSDMD-KO reduces lung inflammation in hyperoxia-exposed lungs. (**A**) GSDMD expression was detected by immunostaining (brown signals) on lung sections. GSDMD was expressed in alveolar septa in RA-exposed WT (WT-RA) and hyperoxia-exposed WT (WT-O_2_) lungs. GSDMD was also detected in the infiltrating cells in the alveolar airspaces of WT-O_2_ lungs. GSDMD was undetectable in RA-exposed knockout (KO-RA) and hyperoxia-exposed KO (KO-O_2_) lungs. (**B**) qRT-PCR showed hyperoxia-upregulated GSDMD gene expression in the WT lungs, but it was barely detectable in the GSDMD-KO lungs. n = 4/group. (**C**) Immunostaining for Mac-3 (a macrophage marker, brown signals, red arrows). (**D**) There was a significant increase of macrophage infiltration into the alveolar airspaces in the WT-O_2_ group compared to the WT-RA group, but KO-O_2_ had reduced macrophage infiltration compared to the WT-O_2_ group. (**E**) Immunostaining for neutrophil elastase (NE, a marker for neutrophils, brown signals, red arrows). (**F**) Neutrophil count was increased by hyperoxia in the WT lungs, while hyperoxia-exposed GSDMD-KO lungs had reduced neutrophil infiltration compared to hyperoxia-exposed WT lungs. n = 5/group. *P < 0.05, ***P < 0.001, WT-RA vs WT-O_2_. ^#^*P* < 0.05, ^###^*P* < 0.001, WT-O_2_ vs KO-O_2_. 20 × magnification. Scale bars: 50 mm.

We first showed that GSDMD is extensively expressed in the alveolar septa of both RA and hyperoxia-exposed WT lungs but undetectable in both RA and hyperoxia-exposed GSDMD-KO lungs (Fig. 1A). GSDMD was also highly expressed in the infiltrating cells in the alveolar airspaces of hyperoxia-exposed WT lungs (Fig. 1A). Quantitative RT-PCR (qRT-PCR) confirmed that GSDMD gene expression was increased by hyperoxia in the WT lungs and that GSDMD gene expression was barely detectable in RA or hyperoxia-exposed GSDMD-KO lungs (Fig. 1B). We next examined lung sections for macrophage and neutrophil infiltration by immunostaining to assess whether GSDMD-KO affects hyperoxia-induced lung inflammation. Histologically, there were many infiltrated macrophages in the WT hyperoxia-exposed lungs compared to the other three groups (Fig. 1C). Quantitative analysis showed macrophage count was 4-fold higher than the other three groups (Fig. 1D). Similarly, there were many infiltrated neutrophils in hyperoxic WT lungs (Fig. 1E), and their count was 8-fold higher than the other three groups (Fig. 1F). Thus, GSDMD deficiency ameliorated hyperoxia-induced lung inflammation.

### GSDMD deficiency improves alveolarization in hyperoxia-exposed lungs

**Figure 2:**
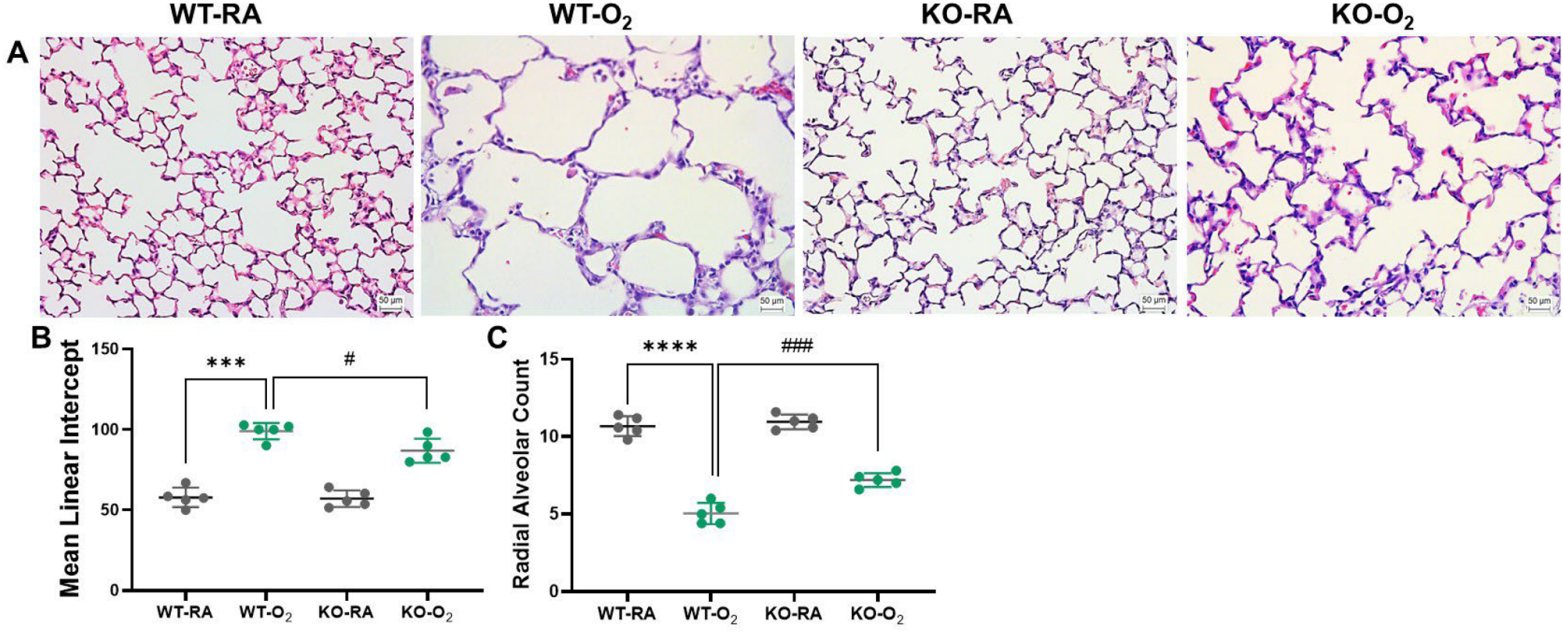
(**A**) Representative H&E stained lung tissue sections from WT-RA, WT-O_2_, KO-RA, and KO-O_2_ mice. Larger and simplified alveolar structures were observed in the lungs of the WT-O_2_ group. (**B**) Alveolarization was assessed by counting MLI, which showed an increased MLI in the WT-O_2_ group, while the GSDMD-KO group exposed to hyperoxia had a decreased MLI. (**C**) RAC was decreased in the hyperoxia-exposed WT lungs but increased in the hyperoxia-exposed GSDMD-KO lungs compared to hyperoxic WT lungs. n = 5/group. ***P < 0.001, ****P < 0.0001, WT-RA vs WT-O_2_. ^#^*P* < 0.05, ^###^*P* < 0.001, WT-O_2_ vs KO-O_2_. 20 × magnification. Scale bars: 50 mm.

Impaired alveolarization is one of the pathological hallmarks of BPD. When examined histologically, the hyperoxia-exposed WT animals showed marked simplification of the alveoli evidenced by larger alveoli, while the GSDMD-KO hyperoxia-exposed group appeared more normal (Fig. 2A). Morphometrical analysis showed a 69% increase in mean linear intercept (MLI) (Fig. 2B) and a 2-fold decreased radial alveolar count (RAC) (Fig. 2C) in the WT hyperoxic group compared to the WT RA-exposed group. In contrast, the MLI was lower (Fig. 2B), and RAC was higher (Fig. 2C) in the GSDMD-KO hyperoxia-exposed group compared to the WT hyperoxia-exposed group. These results confirm improved alveolarization in GSDMD-KO hyperoxia-exposed lungs.

### GSDMD deficiency improves vascularization and reduces vascular remodeling in hyperoxia-exposed lungs

**Figure 3:**
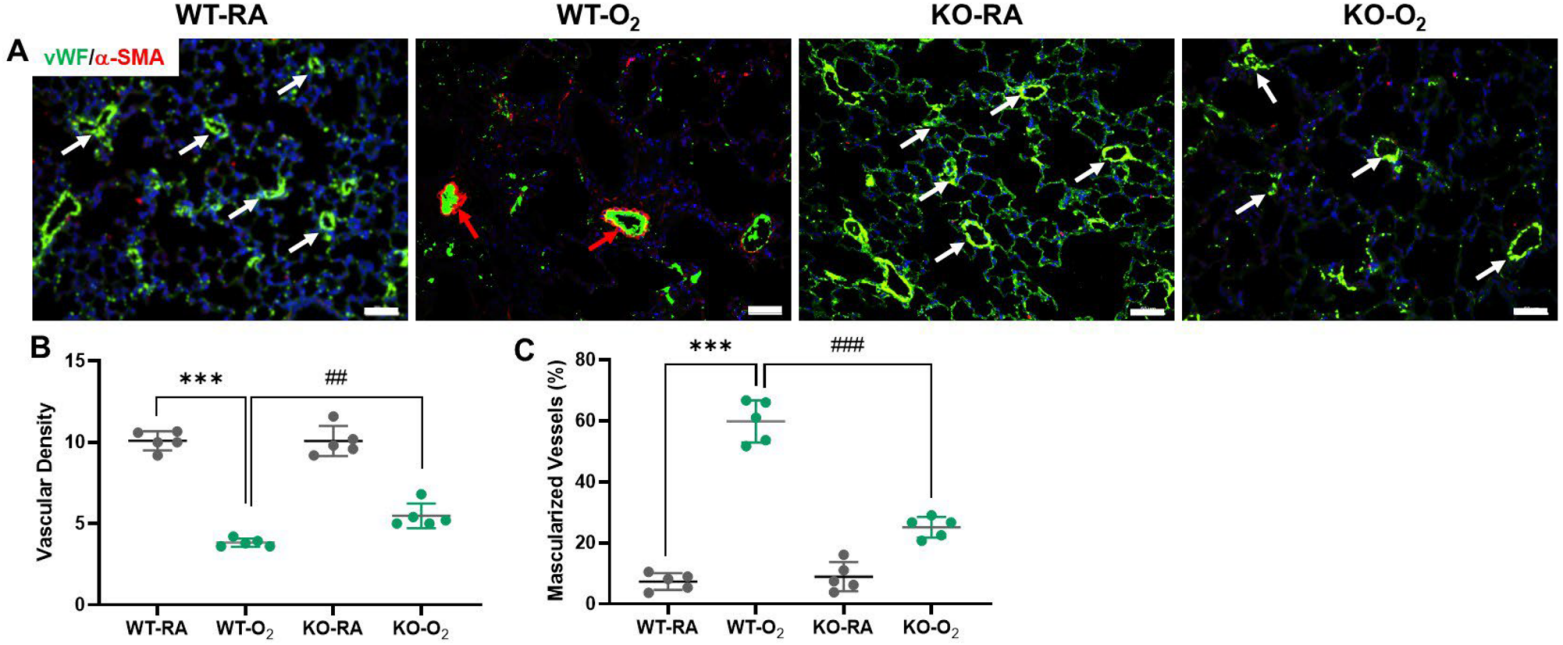
GSDMD-KO improves vascularization and reduces vascular remodeling in hyperoxia-exposed lungs. (**A**) Lung vascularization and vascular remodeling were assessed by double Immunofluorescence for vWF (an endothelial marker, green stain, white arrows), α-SMA (a smooth muscle marker, red stain, red arrows), and DAPI nuclear staining (blue stain). (**B**) Vascular densities (vessels < 50 μm) were decreased in WT-O_2_ lungs but increased in KO-O_2_ lungs compared to the WT-O_2_ group. (**C**) Muscularized vessels (> 50% circumference staining for α-SMA) were increased in WT-O_2_ lungs but less in KO-O_2_ lungs. n = 5/groups. ***P < 0.001, WT-RA vs WT-O_2_. ^##^*P* < 0.01, ^###^*P* < 0.001, WT-O_2_ vs KO-O_2._ 20 × magnification. Scale bars: 50 mm.

Impaired vascularization and pulmonary vascular remodeling are important features of hyperoxia-induced lung injury that are associated with the development of pulmonary hypertension. As illustrated in Fig. 3A,B, the hyperoxia-exposed WT group showed a 62% decrease in vascular density compared to the RA-exposed WT group. However, hyperoxia-exposed GSDMD-KO mice had a 30% increase in vascular density compared to hyperoxic WT mice. The hyperoxia-exposed WT group showed an eight-fold increase in muscularized vessels compared to the RA-exposed WT group (Fig. 3A,C). GSDMD-KO was found to decrease vessel muscularization by more than two-fold (Fig. 3A,C). These results demonstrate that GSDMD-KO improves vascular growth and attenuates vascular remodeling in hyperoxic condition.

### GSDMD deficiency improves cell survival and decreases cell death in hyperoxia-exposed lungs

**Figure 4:**
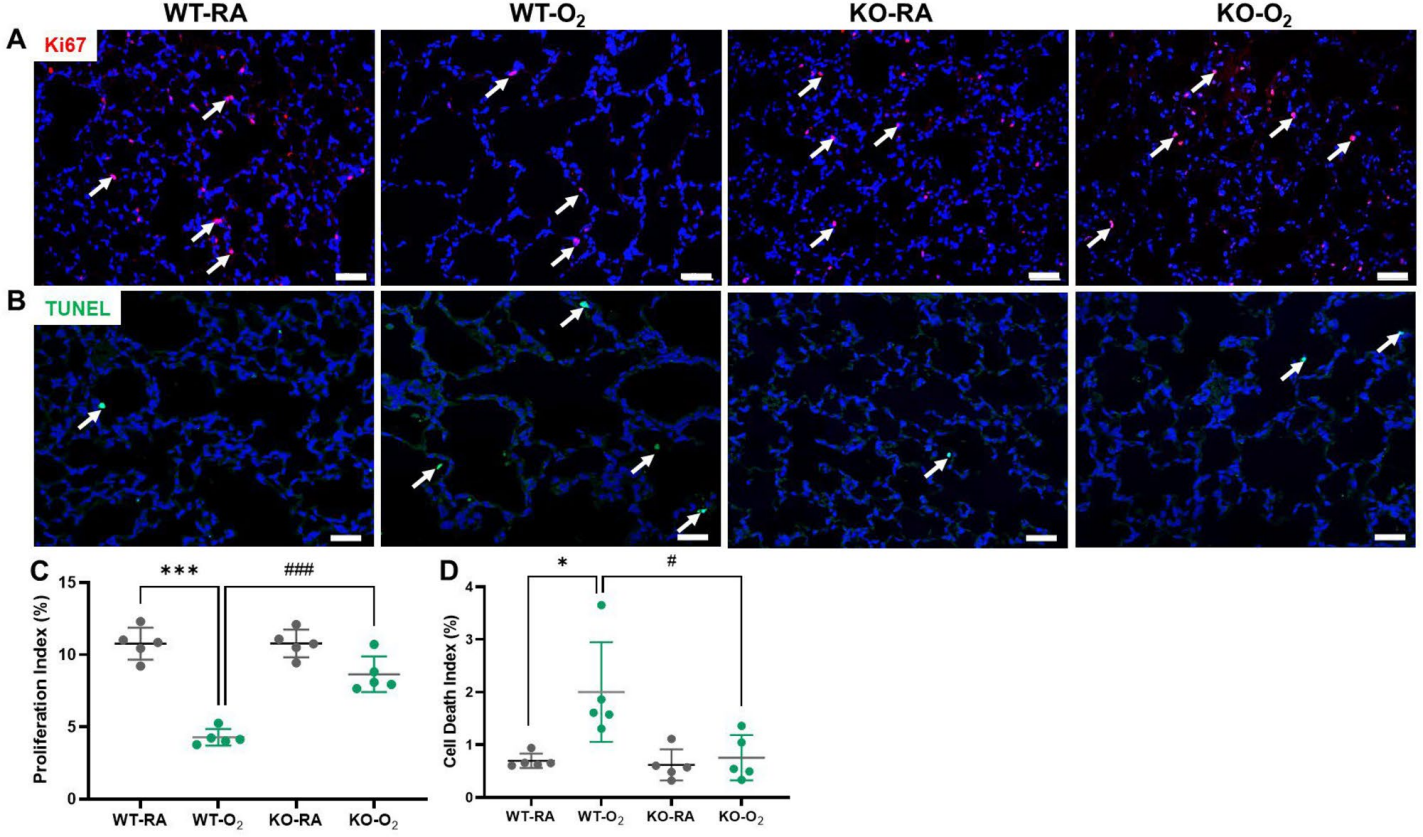
GSDMD-KO improves cell survival in hyperoxia-exposed lungs. (**A**) Immunofluorescent staining for Ki67 (pink signals, white arrows) and DAPI staining (blue signals) were performed to assess cell proliferation. (**C**) Quantification of the cell proliferation index (percentage of Ki67 positive nuclei divided by total nuclei) revealed that hyperoxia decreased cell proliferation in WT lungs but GSDMD-KO improved cell proliferation in hyperoxia-exposed lungs. (**B**) TUNEL Assay (green signals, white arrows) and DAPI nuclear stain (blue signals) were used to identify dead cells. (**D**) Quantification of cell death (percentage of apoptotic nuclei divided by total nuclei) revealed that WT lungs had increased cell death when exposed to hyperoxia. In contrast, hyperoxia-exposed KO lungs had significantly less cell death. n = 5/group. *P < 0.05, ***P < 0.001, WT-RA vs WT-O_2._^#^*P* < 0.05, ^###^*P* < 0.001, WT-O_2_ vs KO-O_2._ 20 × magnification. Scale bars: 50 μm.

GSDMD is a key executor of inflammasome-induced pyroptosis and hyperoxia is known to reduce cell survival and cause cell death in BPD models. We found that our hyperoxia-exposed WT group showed a 60% decrease in cell proliferation compared to the RA-exposed WT group (Fig. 4A,C). However, the GSDMD-KO hyperoxic group was found to have a 77% increased cell proliferation compared to the WT hyperoxia-exposed group (Fig. 4A,C). And when we assessed cell death our data showed that the hyperoxia-exposed WT group had a nearly two-fold increase in cell death compared to the RA-exposed WT group (Fig. 4B,D), while the GSDMD-KO hyperoxia-exposed group was found to have more than two-fold less cell death than the WT hyperoxia-exposed group (Fig. 4B,D).

### GSDMD deficiency ameliorates hyperoxia modulation of inflammatory, tissue remodeling, and developmental gene pathways in neonatal lungs

**Figure 5:**
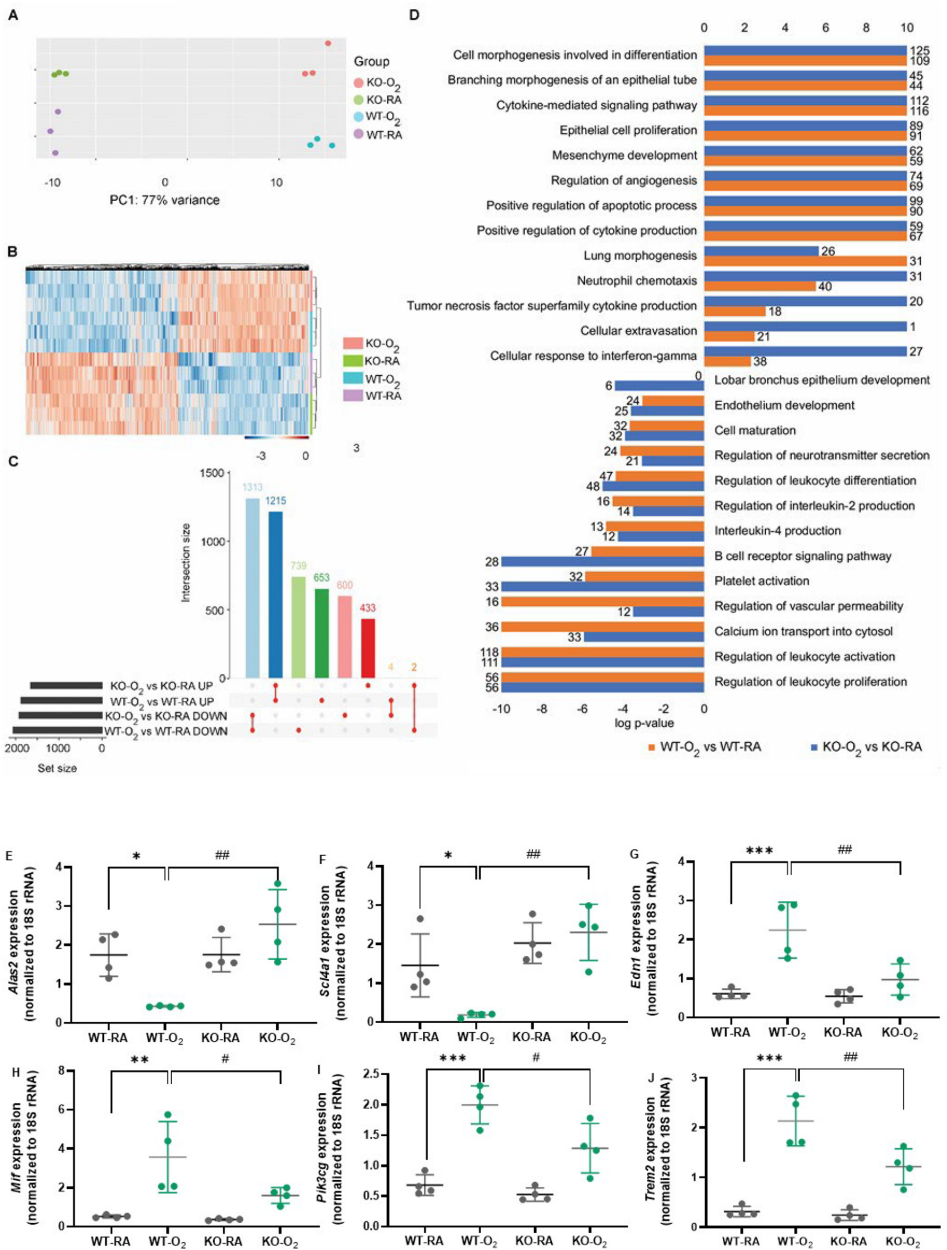
GSDMD-KO reduces hyperoxia modulation of inflammatory, tissue remodeling, and developmental pathways in the neonatal lung. (**A**) Principal component analysis (PCA) plot showing separation of WT and GSDMD-KO mice by PC1 and RA and O_2_ animals by PC2. (**B**) Heatmap of differentially expressed genes in WT-O_2_ vs WT-RA and KO-O_2_ vs KO RA. (**C**) UpSet plot showing overlap of genes differentially regulated by hyperoxia in WT and GSDMD-KO lungs. Hyperoxia modulated the expression of 3926 genes in WT and 3567 genes in GSDMD-KO lungs. (**D**) Overrepresentation analysis using Toppcluster to identify similarities and dissimilarities of Gene Ontology terms and pathways modulated by hyperoxia in WT and GSDMD-KO lungs. Bars represent the log P-value, and the number of genes associated with each term is displayed at the end of the bar. In GSDMD-KO lungs, genes induced by hyperoxia were more strongly associated with TNF superfamily cytokine production, cellular extravasation, and cellular response to IFN-γ, while suppressed genes in GSDMD-KO were uniquely associated with lobar bronchus epithelium development and cell receptor signaling pathways. n = 3 animals/group. qRT-PCR validation of differentially expressed genes between hyperoxia-exposed WT and hyperoxia-exposed GSDMD-KO lungs included *Alas2* (**E**), *Scl4a1* (**F**), *Edn1* (**G**), *Mif* (**H**), *Pik3cg* (**I**), and *Trem 2* (**J**). n = 4/group. *P < 0.05, **P < 0.01, ***P < 0.001, WT-O_2_ vs WT-RA. ^#^*P* < 0.05, ^##^*P* < 0.01, WT-O_2_ vs KO-O_2_.

To understand how GSDMD-KO affects the lung response to hyperoxia at the transcriptome level, RNA-seq was performed on whole lung RNA extracts from the four experimental groups. Principal component analysis of the lung transcriptomes showed clear separation for the RA and hyperoxia-exposed animals along principal component (PC) 1, which was responsible for 77% of the variance, and separation of WT and GSDMD-KO along PC2 (Fig. 5A). In order to identify the similarities and divergencies in genes and biological processes modulated by hyperoxia in WT compared to GSDMD-KO animals we first performed differential expression analysis comparing RA-exposed WT vs hyperoxia-exposed WT lungs and RA-exposed GSDMD-KO vs hyperoxia-exposed GSDMD-KO lungs and compared results at the gene and pathway levels (Fig. 5B). In WT animals, hyperoxia differentially regulated 3926 genes, and in GSDMD-KO animals hyperoxia differentially regulated 3567 genes. There were 2528 genes commonly induced or suppressed by hyperoxia in both WT and GSDMD-KO lungs and 1392 genes uniquely regulated in WT lungs, and 1033 genes uniquely regulated in GSDMD-KO lungs (Fig. 5C). We performed an overrepresentation analysis on Toppcluster to identify biological processes and pathways commonly and uniquely associated with hyperoxia-regulated genes in WT and GSDMD-KO animals. As illustrated in Fig. 5D, in GSDMD-KO animals genes induced by hyperoxia were more strongly associated with neutrophil chemotaxis, TNF signaling, cellular extravasation, and cellular response to interferon. In WT animals genes induced by hyperoxia were more strongly associated with lung morphogenesis and genes suppressed by hyperoxia were more associated with the regulation of vascular permeability and calcium ion transport into the cytosol.

We performed qRT-PCR to validate some of the genes that were differentially up or downregulated by hyperoxia in WT and GSDMD-KO lungs. Mitochondrial erythroid-specific 5-aminolevulinate synthase 2 (*Alas2*) (Fig. 5E) and solute carrier family 4 member 1 (*Slc4a1*) (Fig. 5F) were downregulated by hyperoxia in the WT lungs, but they were upregulated in hyperoxia-exposed GSDMD-KO lungs compared to hyperoxia-exposed WT lungs. Endothelin 1 (*Edn1*) (Fig. 5G), macrophage migration inhibitory factor (*Mif*) (Fig. 5H), phosphatidylinositol 3-kinase, catalytic subunit gamma (*Pik3cg*) (Fig. 5I) and triggering receptor expressed on myeloid cells 2 (*Trem2*) (Fig. 5J) were upregulated by hyperoxia in the WT lungs. However, they were downregulated in hyperoxia-exposed GSDMD-KO lungs compared to hyperoxia-exposed WT lungs.

Cluster comparison of analysis using ClusterProfiler showed that genes induced by hyperoxia in WT animals but not in GSDMD-KO animals were associated with tubulin binding, DNA replication origin binding, epidermal growth factor receptor (EGFR) binding, cysteine-endopeptidase regulator activity involved in apoptotic process, and beta-catenin (β-catenin) binding (Supplemental Fig. 1). Genes induced by hyperoxia in GSDMD-KO lungs but not in WT lungs were associated with nucleotide receptor activity, amino acid binding, retinal dehydrogenase activity, MAP kinase phosphatase activity, and LRR domain binding. Genes downregulated by hyperoxia in WT lungs but not in GSDMD-KO lungs were associated with oxygen carrier activity, ligand-gated ion channel activity, MHC protein binding, and fibroblast growth factor (FGF) binding. Genes downregulated by hyperoxia in GSDMD-KO lungs but not in WT lungs were associated with protein phosphorylated amino acid binding, endonuclease activity, monooxygenase activity, and Toll-like receptor (TLR) binding (Supplemental Fig. 1). Direct comparison of the lung transcriptomes of hyperoxia-exposed GSDMD-KO mice to hyperoxia-exposed WT mice revealed 53 differentially regulated genes with 27 genes induced and 26 genes suppressed in GSDMD-KO mice (Supplemental Fig. 2A). Genes preferentially induced by hyperoxia in GSDMD-KO lungs relative to WT lungs were associated with homeostasis of the number of cells, reactive oxygen species metabolic process, response to oxidative stress, gas transport, and regulation of leukocyte proliferation (Supplemental Fig. 2B). Genes preferentially suppressed by hyperoxia in GSDMD-KO were associated with metallopeptidase activity, stress-induced cell death, retinol binding, and wide-pore channel activity (Supplemental Fig. 2C).

To further identify genes that were modulated by GSDMD-KO in the setting of hyperoxia we performed interaction analysis using the likelihood ratio test on DESeq2, which revealed 1108 genes using a significance threshold of FDR < 0.1 and fold change > 1. Overrepresentation analyses for Gene Ontology term showed that genes regulated by GSDMD-KO in the setting of hyperoxia were associated with homeostasis of number of cells, epithelial tube morphogenesis, organ growth, epithelial cell proliferation, regulation of inflammatory response, regulation of macrophage activation, and regulation of endothelial cell proliferation, etc. (Supplemental Fig. 3A). Overrepresentation analysis for KEGG pathways showed that GSDMD-KO modulated cytokine-cytokine receptor interaction, PI3K-Akt signaling pathway, IL-17 signaling pathway, TNF signaling pathway, NF-kappaB (NF-κB) signaling pathway, cAMP signaling, and Wnt signaling pathway, etc. (Supplemental Fig. 3B). Network plot of the top 5 biological processes enriched for among genes regulated by GSDMD-KO in hyperoxia were illustrated in Supplemental Fig. 3C. Overall, the findings from the transcriptome analyses suggest that GSDMD-KO animals were relatively resistant to hyperoxic lung damage compared to WT animals because they were able to better regulate the inflammatory response through regulation of cytokine-cytokine receptor interaction, cAMP, TNF, IL-17 and NF-κB signaling; oxidative stress-induced cell death; and the tissue remodeling response through regulation of cell adhesion molecules. Moreover, GSDMD-KO also modulated Wnt and PI3K/Akt signaling pathways that are important in lung development and repair.

### GSDMD expression in retinas

**Figure 6:**
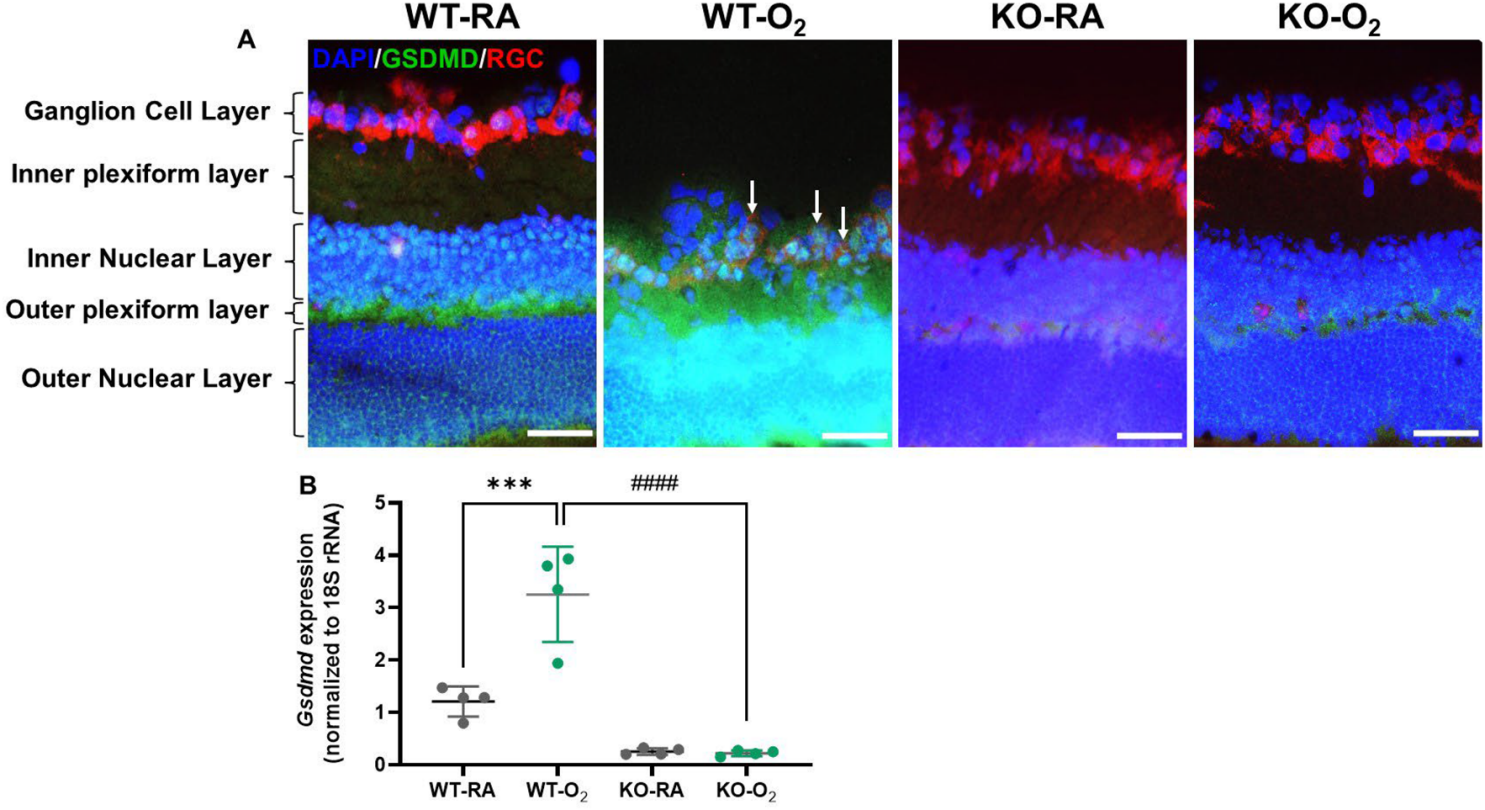
GSDMD expression in the retina. (**A**) Double immunofluorescence staining for GSDMD (green signals) and RBPMS (an RGC marker, red signals) and DAPI nuclear stain (blue signals) were performed to assess GSDMD expression and colocalize it with RGC. GSDMD was detected in the outer plexiform layer in the WT-RA retinas. GSDMD expression was increased in the outer plexiform layer and ganglion cell layer with some colocalized in the RGC (white arrows, orange signals) in the WT-O_2_ retinas. There was decreased GSDMD expression in the outer plexiform layers in KO-RA and KO-O_2_ retinas. (**B**) qRT-PCR showed hyperoxia-upregulated GSDMD gene expression in the WT retinas, but it was barely detectable in the GSDMD-KO retinas. n = 4/group. 20 × magnification. Scale bars: 50 μm.

We examined GSDMD protein expression and colocalized it with a retinal ganglion cell (RGC) marker, the RNA binding protein with multiple splicing (RBPMS)^17^ in retina cross-sections. In hyperoxia-exposed WT retinas, GSDMD expression was increased in the outer plexiform layer, and what remains of the inner nuclear layer, and the RGC layer (white arrows) which was visibly thinner and disorganized and positioned next to the outer plexiform layer due to the thinning of the inner plexiform layer and inner nuclear layer. GSDMD expression was undetectable in RA-exposed GSDMD-KO and hyperoxia-exposed GSDMD-KO retinas (Fig. 6A). GSDMD gene expression was assessed by qRT-PCR, which showed that it was upregulated by hyperoxia in the WT retinas (Fig. 6B), but it was barely detectable in the GSDMD-KO retinas (Fig. 6B).

### GSDMD deficiency prevents hyperoxia-induced retinal vasoobliteration and neovascularization

**Figure 7:**
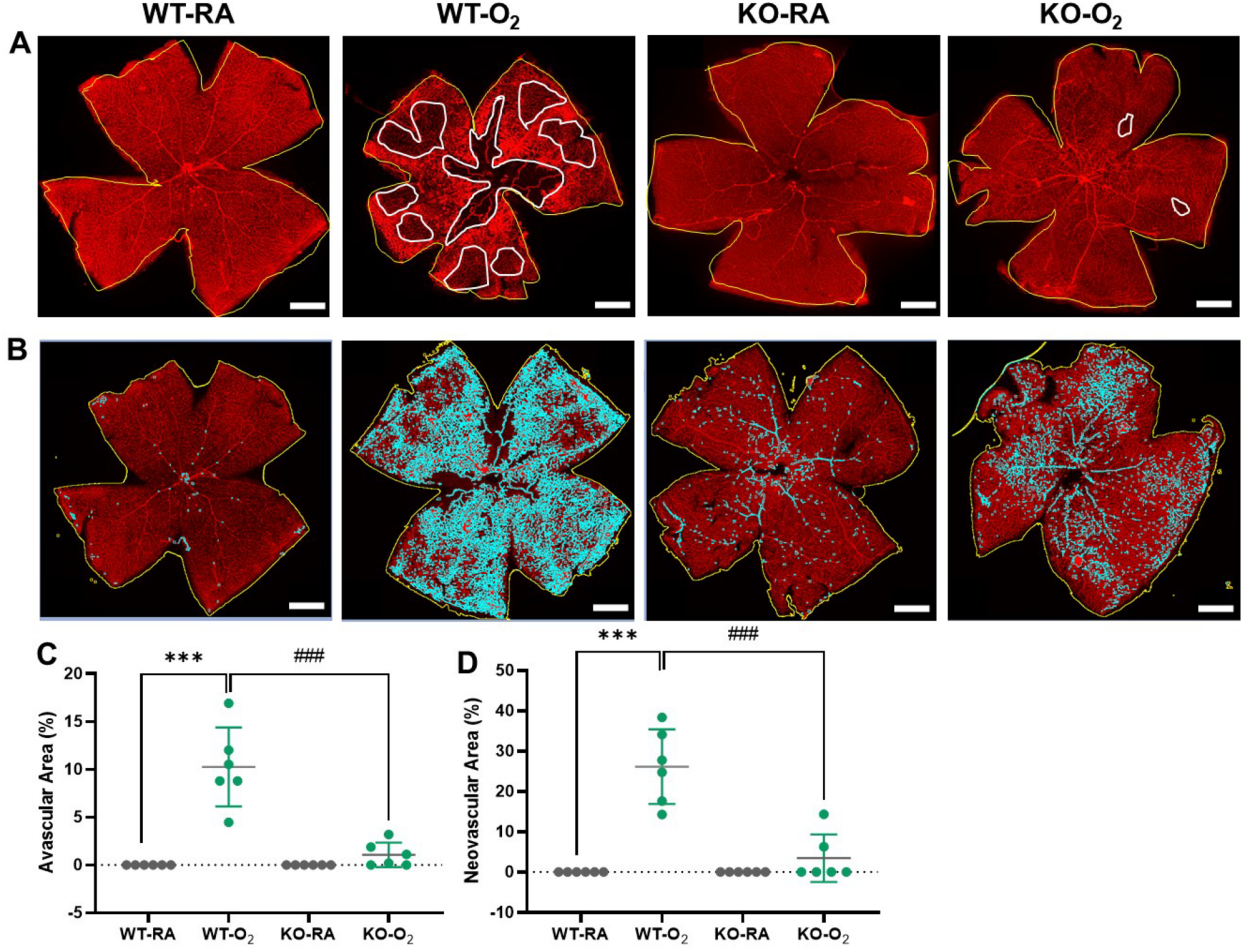
GSDMD deficiency prevents hyperoxia-induced retinal vasoobliteration and neovascularization. Immunofluorescent staining for isolectin (red signals) was used to identify retinal vessels. (**A**) The total retina area and total avascular area were outlined in yellow and white, respectively. (**B**) The total retinal area was outlined in yellow and total neovascular area was measured via isolectin intensity (cyan signals). (**C**) Quantification of the percentage of the avascular area (avascular area divided by total retinal area) showed that retinas from hyperoxia-exposed WT mice had markedly increased vasoobliteration. In contrast, GSDMD-KO mouse retinas had minimal vasoobliteration after hyperoxia exposure. (**D**) Quantification of the percentage of the neovascular area (neovascular area divided by total retinal area) demonstrated that retinas from hyperoxia-exposed WT mice had greatly increased neovascularization, but GSDMD-KO mouse retinas had comparatively little neovascularization when exposed to hyperoxia. n = 6/group. ***P < 0.001, WT-RA vs WT-O_2._^###^*P* < 0.001, WT-O_2_ vs KO-O_2._ 5 × magnification. Scale bars: 50 μm.

Hyperoxia-induced rodent models of ROP replicate the biphasic pathological characteristics of vasoobliteration and neovascularization of human ROP. Oxygen exposure from P7 to P12 results in vasoobliteration of the central retinal vasculature^18,19^. Then during the RA recovery from P12-P17, neovessels form at the junction between the vascularized and non-vascularized retinas^18,19^. When we investigated whether GSDMD-KO attenuates these two hyperoxia-induced pathological changes we found that the hyperoxia-exposed WT mice had a more than a 16-fold increase in avascular area compared to RA-exposed WT retinas (Fig. 7A,C). In stark contrast, the hyperoxia-exposed GSDMD-KO mice had a marked decrease (77%) in vasoobliterated area compared to the hyperoxic WT group (Fig. 7C). When we investigated if GSDMD-KO influences retinal neovascularization we found that the hyperoxia-exposed WT mice had increased retinal neovascularization compared to WT RA-exposed mice as shown in Fig. 7B. In vivid contrast, the GSDMD-KO mice were largely resistant to hyperoxia as the area of neovascularization induced by hyperoxia was nearly eight-fold less than that induced in WT mice (Fig. 7D). Thus, knockout of GSDMD prevents hyperoxia-induced ROP-like pathology.

### GSDMD deficiency prevents hyperoxia-induced retinal thinning

**Figure 8:**
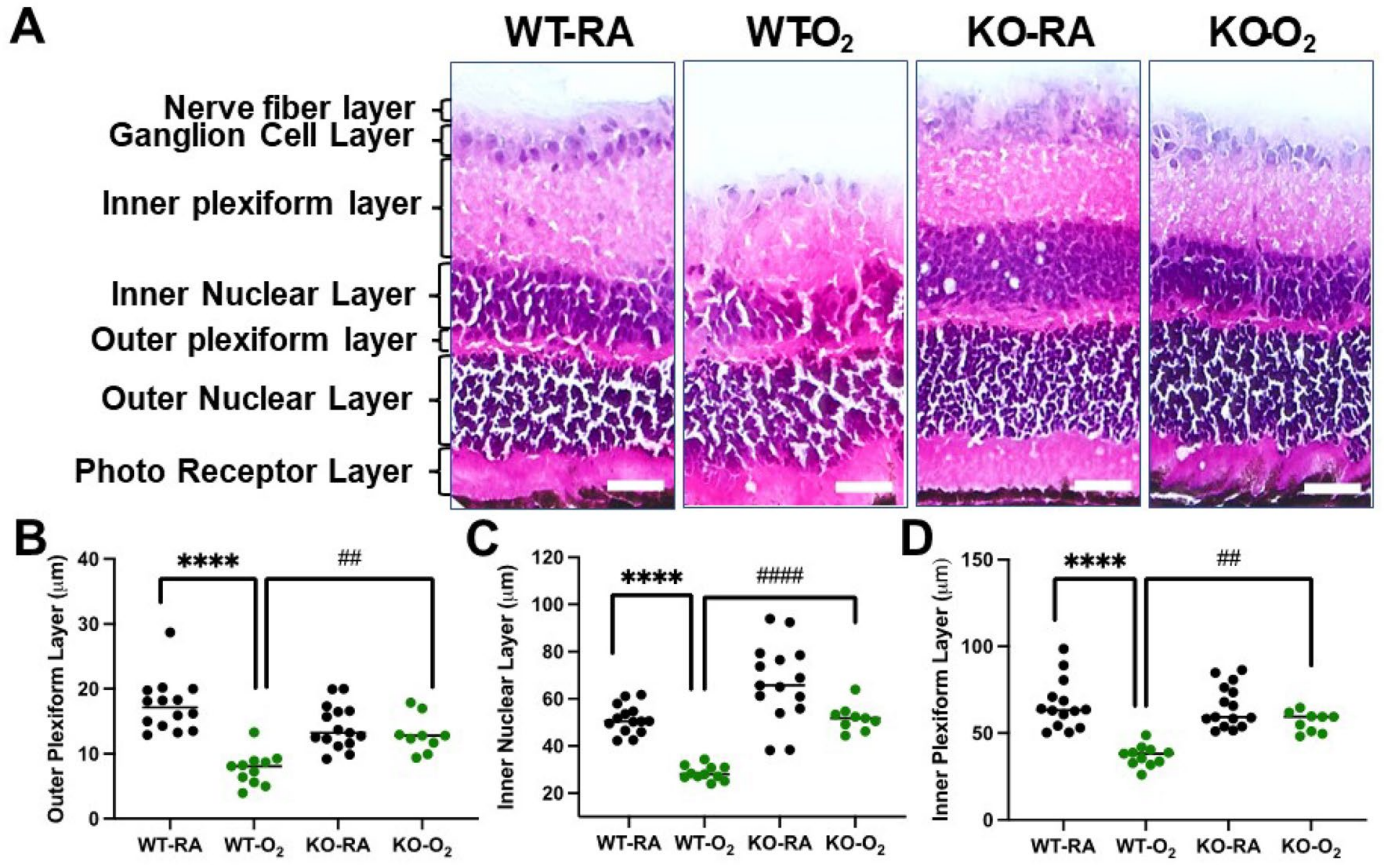
GSDMD-KO prevents hyperoxia-induced retinal thinning. (**A**) The thickness of the individual retinal layer was assessed on H&E stained eye sections. Hyperoxia exposure reduced the thicknesses of the outer plexiform layer, inner plexiform layer, and inner nuclear layer in WT mice. However, GSDMD-KO mice had overall decreased retinal thinning in these three layers compared to the WT hyperoxic group. (**B**) Outer plexiform layer: ****P < 0.0001, WT-RA vs WT-O_2_, ^##^*P* < 0.01, WT-O_2_ vs KO-O_2._ **C.** Inner plexiform layer: ****P < 0.0001, WT-RA vs WT-O_2_, ^####^*P* < 0.01, WT-O_2_ vs KO-O_2_. (**D**) Inner nuclear layer: ****P < 0.0001, WT-RA vs WT-O_2_. ^##^*P* < 0.01, WT-O_2_ vs KO-O_2._ n = 9–15/group. 20 × magnification. Scale bars: 50 μm.

The vertebrate retina is a layered structure with a large diversity of component cells that form morphologically and functionally distinct circuits that work in parallel, and in combination, to produce a complex visual output^20^. We performed measurements of the thickness of each specified layer. Overall retinal thinning in the WT hyperoxia group compared to the WT-RA group can be appreciated in the shown histology (Fig. 8A). We found that the GSDMD-KO group had overall decreased retinal thinning compared to the WT hyperoxia group, particularly seen in the outer plexiform (Fig. 8B), inner plexiform (Fig. 8C) and inner nuclear layers (Fig. 8D). There is also significant ablation of the ganglion cell layer in the WT-O_2_ group as seen in Fig. 8A (data not quantitated).

### Knockout of GSDMD reduces hyperoxia-induced retinal inflammation

**Figure 9:**
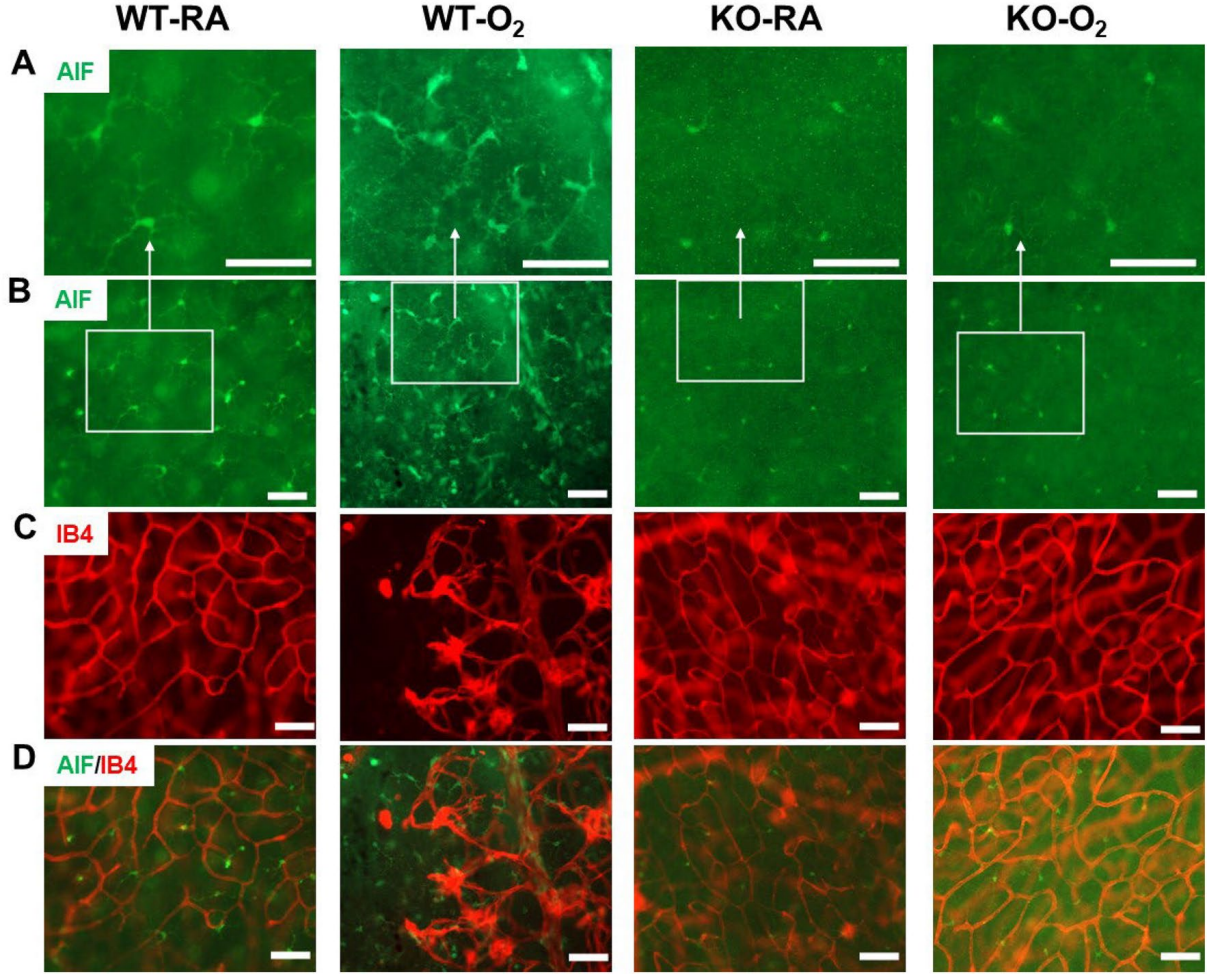
GSDMD-KO prevents hyperoxia activation of microglia in the retina. Double Immunofluorescent staining for allograft inflammatory factor 1 (AIF1), a microglial marker (green signals), and IB4, a vascular marker (red signals), was performed to assess microglial expression and activation in retinas. (**A**,**B**) The microglia cells in the WT-O_2_ retinas were disorganized and had enlarged bodies and dendrites compared to retinas from WT-RA, KO-RA, and KO-O_2_ mice. (**A**) Representative focal enlarged areas of (**B**) in the white boxes. (**C**) The vessels in the WT-O_2_ retinas were less, blunt, and disorganized compared to the other three groups. (**D**) Merging of (**B**) and (**C**) showed that activated microglia were in abundance in the avascular area of the retinas from WT-O_2_ mice. 20 × magnification. Scale bar: 50 μm.

We next examined the retinas for inflammation by immunofluorescent staining for allograft inflammatory factor-1 (AIF), a marker of microglia activation^21^. In hyperoxia-exposed WT retinas, there were increased numbers of activated microglia cells that had a reduction in the territory, enlargement of the cell body, and a more irregular cell shape as well as a self-association for each other (Fig. 9A,B). In contrast, the retinas from the other three groups had fewer microglia cells which had small cell bodies, and few dendrites and were evenly disbursed throughout the retinas. Furthermore, when AIF1 stained sections were dual stained with isolectin, we found abundant activated microglia cells in areas of vasoobliteration and neovascularization (Fig. 9C,D). These results highlight that GSDMD-KO prevents hyperoxia-induced retinal inflammation.

### GSDMD deficiency reduces hyperoxia modulation of inflammatory, cell death, and vascular and visual developmental gene pathways in the neonatal retinas

**Figure 10:**
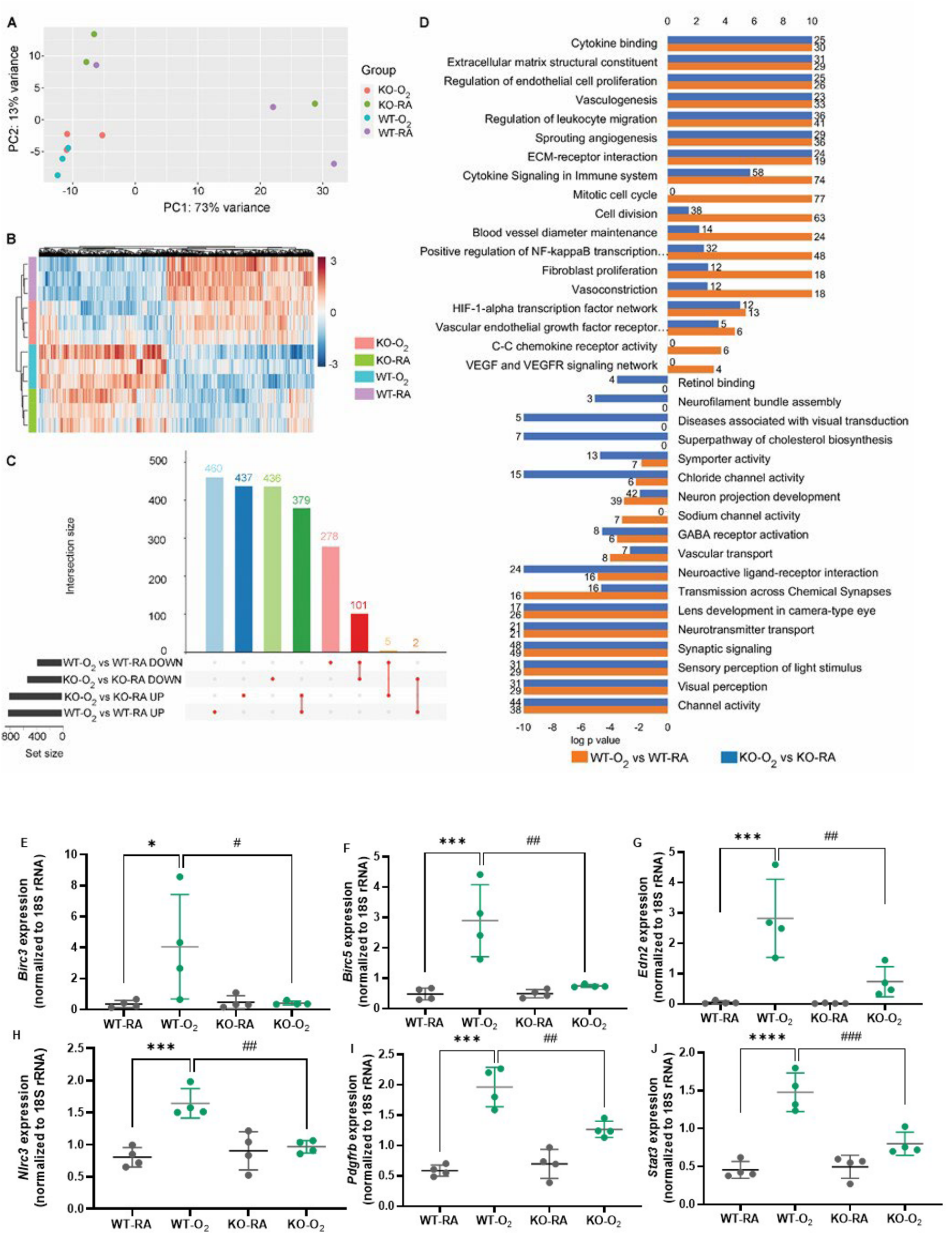
GSDMD-KO reduces the modulation of inflammatory, tissue remodeling, and angiogenic pathways by hyperoxia in the neonatal retina. (**A**) PCA plot showing separation of RA and O_2_ animals with overlap of WT and GSDMD-KO animals. (**B**) Heatmap of differentially expressed genes in WT-O_2_ vs WT-RA and KO-O_2_ vs KO-RA. (**C**) UpSet plot showing overlap of genes differentially regulated by hyperoxia in WT and GSDMD-KO retinas. In WT retinas, hyperoxia regulated the expression of 1224 genes, and in GSDMD-KO retinas hyperoxia regulated 1360 genes. (**D**) Overrepresentation analysis using Toppcluster to identify similarities and dissimilarities of biological processes modulated by hyperoxia in WT and GSDMD-KO retinas. Bars represent the log *P*-value, and the number of genes associated with each term is displayed at the end of the bar. In WT retinas, genes induced by hyperoxia were strongly associated with cellular division, blood vessel diameter maintenance, positive regulation of NF-κB transcription, fibroblast proliferation, vasoconstriction, and VEGF and VEGFR signaling network than in GSDMD-KO retinas. Suppressed genes were uniquely associated with retinol binding, neurofilament bundle assembly, diseases associated with visual transduction, and neuroactive ligand-receptor interaction in GSDMD-KO retinas. n = 3 animals/group. qRT-PCR validated six genes that were upregulated by hyperoxia in the WT retinas, but they were downregulated in KO-O_2_ retinas compared to WT-O_2_ retinas including *Birc3* (**E**), *Birc5* (**F**), *Edn2* (**G**), *Nlrc3* (**H**), *Pdgfrb* (**I**), and *Stat3* (**J**). n = 4/group. *P < 0.05, **P < 0.01, ***P < 0.001, ****P < 0.0001, WT-O_2_ vs WT-RA. ^#^*P* < 0.05, ^##^*P* < 0.01, ^###^*P* < 0.001, WT-O_2_ vs KO-O_2_.

To better characterize the effects of GSDMD-KO and identify genes modulated by GSDMD-KO on hyperoxia-induced retinal injury, we performed RNA-seq of whole retinas. Principal component analysis of the retina transcriptome showed clustering of hyperoxia-exposed GSDMD-KO and WT animals (Fig. 10A). To identify similarities and divergencies in genes differentially regulated by hyperoxia in the WT and GSDMD-KO animals, differential expression analysis was performed comparing RA-exposed WT vs hyperoxia-exposed WT retinas and RA-exposed GSDMD-KO vs hyperoxia-exposed GSDMD-KO retinas, and the results were analyzed at the gene expression and pathway levels (Fig. 10B). In WT retinas hyperoxia differentially regulated 1224 genes, and in GSDMD-KO retinas, hyperoxia differentially regulated 1360 genes. There were 480 genes commonly induced or suppressed by hyperoxia in both WT and GSDMD-KO retinas, 738 genes uniquely regulated in WT retinas, and 873 genes uniquely regulated in GSDMD-KO retinas (Fig. 10C). Overrepresentation analysis of genes differentially regulated by hyperoxia in WT and GSDMD-KO retinas showed that in WT retinas, genes induced by hyperoxia were strongly associated with mitotic cell cycle, cellular division, blood vessel diameter maintenance, positive regulation of NF-κB transcription, fibroblast proliferation, vasoconstriction, C–C chemokine receptor activity, and VEGF and VEGFR signaling network (Fig. 10D). In GSDMD-KO retinas, hyperoxia suppressed genes were more uniquely associated with retinol binding, neurofilament bundle assembly, diseases associated with visual transduction and neuroactive ligand-receptor interaction (Fig. 10D).

We performed qRT-PCR to validate some of the differentially regulated genes by hyperoxia in the WT and GSDMD-KO retinas. Hyperoxia upregulated gene expression of baculoviral IAP Repeat Containing 3 (*Birc3*) (Fig. 10E), *Birc5* (Fig. 10F), *Edn2* (Fig. 10G), NLR family CARD-containing 3 (*Nlrc3*) (Fig. 10H), platelet-derived growth factor receptor beta (*Pdgfrb*) (Fig. 10I), and signal transducer and activator of transcription 3 (*Stat3*) (Fig. 10J) in the WT retinas. However, these genes were downregulated in hyperoxia-exposed GSDMD KO retinas compared to hyperoxic WT retinas.

Cluster comparison of analysis using ClusterProfiler showed that genes induced by hyperoxia were associated with chemokine receptor activity, transforming growth factor beta (TGF-β) activated receptor activity, death receptor activity, complement receptor activity, and TNF receptor superfamily binding in WT animals but not in KO animals. Genes suppressed by hyperoxia were associated with glutamate binding, amino acid binding, gap junction channel activity, and cell adhesion mediator activity in WT but not in GSDMD-KO retinas (Supplemental Fig. 4). In GSDMD-KO retinas, hyperoxia upregulated gene pathways were associated with immunoglobulin binding, extracellular matrix structural constituent, growth factor activity, and mitogen-activated protein kinase binding. Hyperoxia downregulated gene pathways were associated with oxygen binding, dopamine receptor binding, and photoreceptor activity. Direct comparison of hyperoxia-exposed GSDMD-KO mice to hyperoxia-exposed WT mice revealed 1224 differentially regulated genes with 841 induced genes and 383 suppressed genes (Supplemental Fig. 5A). Genes preferentially induced in hyperoxia-exposed GSDMD-KO animals compared to WT hyperoxic animals were associated with cytokine-mediated signaling pathway, leukocyte migration, cell chemotaxis, extracellular matrix organization, I-kappaB kinase/NF-κB signaling, tissue remodeling, and fibroblast proliferation (Supplemental Fig. 5B). Genes preferentially suppressed by hyperoxia in GSDMD-KO retinas were associated with cytokine-cytokine receptor interaction, JAK-STAT signaling pathway, TNF signaling pathway, necroptosis, apoptosis, cellular senescence and p53 signaling pathway (Supplemental Fig. 5C).

To further delineate the effect of GSDMD-KO on the retinal transcriptional response to hyperoxia, we performed interaction analysis using the likelihood ratio test on DESeq2, followed by gene set enrichment analysis of genes that met the significance threshold of FDR < 0.1 and fold change > 1, and identified 2818 genes that were modulated by GSDMD-KO. These genes were associated with the Gene Ontology terms of synapse organization, axonogenesis, leukocyte migration, regulation of angiogenesis, tissue remodeling, visual perception, endothelium development, and blood vessel development (Supplemental Fig. 6A). The KEGG pathways were PI3K-Akt signaling pathway, cAMP signaling pathway, cellular senescence, TNF signaling pathway, NF-κB signaling pathway, FoxO signaling pathway, IL-17 signaling pathway, and HIF-1 signaling pathway (Supplemental Fig. 6B). Network plot of the top 5 biological processes enriched for among genes regulated by GSDMD-KO in hyperoxia were illustrated in Supplemental Fig. 6C. Overall, these findings suggest that GSDMD deficiency prevents hyperoxia-induced ROP not only by reducing cell death but also by modulating inflammatory response, tissue remodeling, and vascular and visual developmental pathways.

## Discussion

BPD and ROP are among the most common complications affecting extremely premature infants, and currently, there are no therapies that are effective and safe for either condition. Many clinical studies indicate that BPD is associated with advanced ROP^6,9^. However, the mechanistic link between hyperoxia, BPD and ROP remains to be explored. In this study we focused our investigations on the mechanistic roles of GSDMD in hyperoxia-induced mouse models of BPD and ROP. We provide evidence, for the first time to the best of our knowledge, that GSDMD deficiency ameliorates hyperoxia-induced histopathological BPD and ROP. We also report the effects of GSDMD-KO on hyperoxia-modulated transcriptomes and distinctive enriched biological pathways in the lungs and retinas.

While various prenatal and perinatal factors can lead to lung injury and BPD development, the lung injury, regardless of the cause, is thought to be largely due to a hyperoxia-induced inflammatory response mediated by macrophages and neutrophils which invade the endothelium and alveolar spaces of premature lungs^22^. Our present study demonstrated that hyperoxia-exposed GSDMD-KO animals had significantly less alveolar macrophage and neutrophil infiltration and, therefore, less inflammation. The hyperoxia-exposed GSDMD-KO mice also had a decreased MLI and increased RAC compared to their WT counterparts, demonstrating an improvement in alveolarization and gas exchange surface area. Furthermore, we looked to see if GSDMD-KO had effects on poor vascular growth, another hallmark of BPD. We focused on measuring the vascular density and muscularization of the lung vasculature in our models. Our results demonstrated that GSDMD-KO mice had improved vascularization and less vascular remodeling/muscularization compared to the WT mice under hyperoxic condition. We sought to understand if GSDMD played a role in cell death and cell proliferation through its role in the inflammasome pathway. We found that GSDMD-KO mice were relatively resistant to the hyperoxia-induced lung cell death and depressed cell proliferation present in the WT mice. Overall, these results are consistent with improvements in hyperoxia-induced lung injury, and deranged alveolar and vascular development seen in BPD.

Our lung RNA-seq findings reveal that hyperoxia-induced lung structural damage is associated with the altered expression of many gene pathways that impact normal lung structure and development in the WT lungs that are not similarly altered in GSDMD-KO animals. In the WT lungs, hyperoxia-regulated genes were associated with important developmental pathways such as tubulin binding, EGFR binding, β-catenin binding, oxygen carrier activity and oxygen binding, and FGF binding. However, these gene pathways were not observed to be associated with genes regulated by hyperoxia in GSDMD-KO mice suggesting they are GSDMD dependent. EGF signaling is important for alveolar regeneration, as the administration of recombinant EGF restores alveologenesis and lung inspiratory volume and compliance function in pneumonectomized *Vegfr2*/*Fgfr1* deficient mice^23^. FGF signaling plays key roles not only in early lung development but also in alveolarization by controlling the formation of the elastin extracellular matrix that in turn, guides secondary septa formation leading to the multiplication of alveolar units^24^. Alteration of β-catenin signaling is associated with BPD and idiopathic pulmonary fibrosis in human lungs^25^. Thus, preventing hyperoxia dysregulation of EGF, FGF, and β-catenin signaling pathways may be an important mechanism by which GSDMD-KO improves alveolarization.

When analyzed by Gene Ontology terms, we found many genes were modulated by GSDMD-KO in the lungs exposed to hyperoxia that related to lung development such as homeostasis of cells, epithelial tube morphogenesis and endothelial cell proliferation, organ growth and cell migration, and inflammatory response such as regulation of macrophage activation. Modulation of these pathways by GSDMD-KO may be responsible for improved lung development and reduced lung inflammation. The results from overrepresentation analysis for KEGG pathways identified the most important pathways related to inflammation, tissue remodeling, and organ development that were modulated by GSDMD-KO under hyperoxic condition. The predominant inflammatory pathways identified were the IL-17, TNF, cAMP, and NF-κB signaling pathways. IL-17 plays an important role in instigating and/or exacerbating fetal inflammatory responses that increase neonatal morbidities and mortalities associated with sepsis and BPD^26^. In experimental BPD, IL-17 was shown to be secreted by type 3 innate lymphoid cells, and it can aggravate lung inflammation^27^. High TNF-α levels were detected in preterm infants with BPD^28^ and neonatal mice with experimental BPD^29^. Alveolar macrophage cAMP signaling acts as a key mechanism in tightly controlling TLR4 signaling, thereby dampening inflammatory injury and hence leading to the resolution of lung injury^30^. Activation of NF-κB signaling is associated with hyperoxia-induced BPD in neonatal mice, and its downregulation is associated with improved lung oxidative injury in responding to caffeine treatment^31^. The pathways we identified related to tissue remodeling included metallopeptidase activity and cell adhesion molecules, and imbalance between proteases and their inhibitors are implicated in the pathogenesis of arrested lung alveolarization associated with BPD^32^. Among identified organ development pathways, Wnt signaling plays critical regulatory roles in the function and behavior of the different lung stem cell populations and their niche cells. Especially after lung injury, activated canonical Wnt signaling is crucial for proliferation, survival, and differentiation of lung epithelial stem/progenitor cells^33,34^. Similarly, the PI3K/AKT signaling pathway plays an important role in lung development, as PI3K/AKT signaling in alveolar myofibroblasts regulates alveolarization^35^.

We report six selective genes that have a good correlation between qRT-PCR and RNA-seq results. The *Alas2* gene and *Scl4a1* gene, important in pulmonary vascular remodeling and pulmonary hypertension^36,37^ were only downregulated by hyperoxia in the WT lungs. Genes that were upregulated by hyperoxia in the WT lungs compared to hyperoxia-exposed GSDMD-KO lungs including *Edn1*, *Mif*, *Pik3cg*, and *Trem2*. The *Edn1* gene encodes endothelin 1 which regulates airway smooth muscle remodeling and lung fibroblast proliferation and triggers cytokine storm in human monocytes, which is critical for several inflammatory diseases^38^. MIF is a pleiotropic cytokine produced by various cells, and it acts as a proinflammatory cytokine, which regulates both innate and adaptive immune systems^39^. The *Pik3cg* gene encoded protein p110γ mediates chemokine-induced migration of inflammatory cells, thus playing an important role in initiating inflammatory responses^40^. TREM2 is a receptor expressed on macrophages which drives a gene expression program involved in phagocytosis, lipid catabolism, and energy metabolism^41^. Taken together, modulation of these important genes and pathways regulating inflammatory, tissue remodeling, and organ developmental pathways by GSDMD-KO could contribute to the reduced inflammation, suppressed tissue remodeling, and improved alveolar and vascular development seen in the GSDMD-KO mice after hyperoxia exposure.

Premature neonates also have immature retinas with underdeveloped retinal vasculature. ROP occurs in two phases: hyperoxia and relative hypoxia. Initial exposure to hyperoxia can cause an arrest in retinal vasculature development, leading to phase two: retinal hypoxia in the setting of this initial vasoobliteration. In this phase, an over-production of VEGF occurs in the retinas and subsequently stimulates neovascularization and unrelenting tortuous retinal vascular growth^5,6^. Inflammatory factors, such as cytokines and chemokines, as well as several growth factors, such as neurotrophins, VEGF, and erythropoietin are thought to contribute in some way to increasing the premature infant’s risk for developing ROP^5^. Therapeutic strategies are also being studied and are currently utilized in clinical practice despite known risks. Intraocular VEGF antagonists have been recently used to treat severe ROP, but they have possible systemic anti-angiogenic side effects that are detrimental to the development of other organs, such as the lung and brain, and they do not correct retinal neuronal injury^8^. Using an oxygen-induced retinopathy mouse model^18,19^, we have demonstrated that GSDMD-KO largely prevents both phases of ROP as GSDMD-KO mice had decreased retinal vasoobliteration and improved retinal vascularization.

Since the pathology of ROP blindness extends beyond the retinal vasculature and into the retina, we sought to understand GSDMD’s role in the retinal layer development. We found that under hyperoxia exposure, GSDMD-KO mice had decreased retinal thinning compared to their WT littermates, with major differences observed in the outer plexiform, inner plexiform, and inner nuclear retinal layers. Although we did not measure the thickness of the ganglion cell layer due to the irregularity of the hyperoxia-exposed WT retina, it appeared that this layer was thin, disorganized, and had fewer ganglion cells, while the other three groups had well-organized ganglion cell layer. At the outer plexiform layer, rod and cone photoreceptors synapse onto bipolar cells, and here is the very first synapse of the retina in which coded visual information diverges into distinct parallel pathways^20^. In the inner plexiform layer, cone bipolar cells contact RGC and amacrine cells, and the RGC axons are the sole output neurons of the retina^20^. Thinning of these layers, particularly the outer plexiform layer and ganglion cell layer was associated with GSDMD expression in hyperoxia-exposed WT retinas. Given the critical role of GSDMD in cell death, it is plausible to suggest that increased cell death is responsible for the poor development of these retinal layers and possible visual function. Thus, our combined data illustrate that GSDMD-KO not only prevents retinal vascular derangement leading to ROP but also prevents damage to the retinal layers themselves which should result in better vision. Clinical studies have shown that children with a history of severe ROP also have reduced retinal nerve fiber layer even at 8 years of age^42^. It is plausible that reducing GSDMD activation would also improve retinal nerve layer development in infants with ROP.

Similar to BPD, we found that GSDMD-KO inhibits hyperoxia-induced retinal inflammation as assessed by reduced numbers of activated microglial macrophages in the retinas. Microglial cells play active roles in maintaining the normal structure and functioning of the retina. In a chronic pre-inflammatory environment, microglia become pathologically activated and release excessive inflammatory mediators that promote retina damage and disease progression^43–46^. Preventing chronic microglial activation by GSDMD-KO would certainly reduce retina injury and progression of ROP caused by chronic hyperoxia exposure.

Our retinal RNA-seq data provide a better characterization of how GSDMD deficiency affects hyperoxia-regulated transcriptomes and biological pathways related to ROP. We found hyperoxia upregulated and downregulated distinctive gene pathways in the WT retinas but not in the GSDMD-KO retinas. In the WT retinas, hyperoxia-induced gene pathways were associated with inflammation, such as cytokine signaling in the immune system, NF-κB transcription, cytokine binding, and TNF receptor superfamily binding; with tissue remodeling, such as C–C chemokine receptor activity and TGF-β-activated receptor activity; with cell death activity; and with vascular development such as blood vessel diameter maintenance, HIF-1α transcription factor network, and VEGF and VEGFR signaling network. TNF-α is known to be produced by retinal microglial cells under hypoxia, and it can induce RGC death and contribute to the breakdown of the blood-retinal barrier^47,48^. NF-κB has been shown to be significantly elevated in a rat model of oxygen-induced ROP^49^. The roles of VEGF in the pathogenesis of clinical ROP and experimental ROP are well documented, and intra-ocular injection of VEGF inhibitors is the most used therapy for preterm infants with ROP^50^. HIF-1α is a key transcription factor for VEGF expression under hypoxia, and suppression of HIF-1α protects against mouse models of ROP^51^. Dysregulation of these pathways would undoubtedly contribute to the pathogenesis of ROP seen in our model. In the WT mice, hyperoxia also suppressed genes associated with neuron projection development and transmission across chemical synapses. Downregulation of these genes could lead to poor visual development related to ROP.

Gene set enrichment analysis demonstrated that hyperoxia-induced and suppressed additional gene pathways in the GSDMD-KO retinas, especially those related to vascular development and cell death. Those related to vascular development include angiogenesis, positive regulation of vascular development, and blood vessel diameter maintenance. Upregulation of these gene pathways by GSDMD-KO may contribute to better vascular development under hyperoxia. Hyperoxia downregulated gene pathways were largely related to cell death including necroptosis, apoptosis, cellular senescence, and p53 signaling. Given that GSDMD is a crucial regulator of inflammatory cell death, downregulating these cell death pathways by GSDMD-KO could lead to better development of retinal layers in our model.

Overrepresentation analysis of Gene Ontology terms for retinal genes modulated by GSDMD-KO in the setting of hyperoxia demonstrated many genes related to retinal vascular and neuronal development were modulated, such as synapse organization, axonogenesis, neurogenesis, angiogenesis, visual perception, and endothelium development. In addition, the genes identified by overrepresentation analysis for KEGG pathways in hyperoxia-exposed GSDMD-KO retinas were associated with developmental pathways such as PI3K/Akt, GAPAergic synapsis, Hippo signaling, and FoxO signaling. The GABAergic synapsis plays a role in visual processing^52^. Hippo signaling blocks mammalian retinal muller cell reprogramming, thus playing a role in vision development^53^. The FoxO family of transcription factors regulates apoptosis in RGC depending on their phosphorylation status by PI3K/Akt and cellular localization^54^. Since these pathways are mainly related to retinal vascular and neuronal development, again modulating their expression by GSDMD deficiency could prevent hyperoxia-induced ROP.

A selective six genes, including *Birc3, Birc5, End2, Nlrc3, Pdgfrb,* and *Stat3*, were shown to be upregulated by hyperoxia in the WT retinas but not in hyperoxia-exposed GSDMD-KO retinas by qRT-PCR that correlated with RNA-seq results. *Birc3* is a gene involved in cytokine signaling in the immune system and positive transcription of NF-κB transcription factor activity. It has been shown to be upregulated in a rat model of retinopathy^55^. *Birc5*, a marker for resident microglia of the retinas and is associated with C–C chemokine receptor activity, cytokine signaling in the immune system, mitotic cell cycle, and vasculogenesis^56^. *Edn2* promotes inflammation, injures the blood-retinal barrier and microglial muller cells, and plays a role in experimental retinopathy^57^. *Nlrc3* is a member of the NLR family and a component of the inflammasomes that play an important role in the pathogenesis of ocular diseases^58^. *Pdgfrb* is one of the receptors for PDGF that regulates vasculogenesis and fibroblast proliferation, and it is implicated in the pathogenesis of ROP^59^. *Stat3* regulates cytokine signaling in the immune system, NF-κB transcription factor activity, and endothelial cell proliferation, and its activation in microglia increases pericyte apoptosis^60^. These data further highlight the importance of GSDMD-KO in protecting against hyperoxia-induced inflammation, abnormal vasculogenesis, and tissue remodeling in the retinas.

We also did an interaction analysis of RNA-seq data in order to discover common pathways dysregulated by hyperoxia in both BPD and ROP models. Among the pathways found, the most striking ones are related to inflammation, such as TNF signaling, IL-17 signaling, cAMP signaling, and NF-κB signaling pathways, all of which are thought to play important roles in the pathogenesis of BPD and ROP. Moreover, GSDMD-KO attenuated these pathways in both BPD and ROP models, which is not surprising given that GSDMD is a key regulator of inflammation. The other pathways regulated by hyperoxia in GSDMD-KO in both lungs and retinas were associated with tissue remodeling, such as cell adhesion molecules and cell adhesion mediator activity, and developmental pathways, such as PI3K/Akt signaling pathway. Since the PI3K/Akt pathway is essential for retinal angiogenesis and alveolarization, positive modulation of this pathway would contribute to the better development of both retinal and alveolar structures.

To the best of our knowledge, this is the first study to demonstrate that GSDMD-KO lessens the injurious effects of hyperoxia on the lung and the retina. Our study has a few limitations. First, lung and retina injury in premature neonates is multifactorial, and in our study, we did not evaluate other contributing factors such as infection, steroid use, or intermittent hypoxia. Studies have shown that intrapartum and postnatal infection-associated inflammation are certain risk factors for developing BPD and ROP^61,62^. Likewise, dysregulated VEGF has been linked to the development of BPD^63,64^ and importantly plays a crucial role in the pathogenesis of ROP. In our study, we did not focus on the VEGF nor identify the role, if any, of GSDMD in the expression or action of VEGF in the lung or the retina of neonatal mice. However, we did present data showing global hyperoxia-induced changes in transcriptomes and biological pathways and how GSDMD-KO affects these changes in both organs, including vascular development. In future studies, it would be important to explore the role GSDMD-KO plays in infection, steroid exposure, and intermittent hypoxia-related BPD and ROP.

In conclusion, the results of this study demonstrate that deficiency of GSDMD largely attenuates the damaging effects of hyperoxia on lung and retina histopathology at structural and cellular levels and link these changes by transcriptome expression analysis. GSDMD-KO results in improved lung alveolarization and vascularization, decreased lung inflammation, and reduced cell death under hyperoxia exposure. These structural changes are associated with biological pathways that better regulate inflammatory responses by modulating oxidative stress and limiting tissue remodeling via inhibition of metallopeptidase activity and cell death. GSDMD-KO also improves retinal vascularization and layer development and decreases retinal inflammation. These histological changes are coupled with biological pathways that prevent cell death and avoid activation of inflammatory pathways, cellular senescence, and developmental pathways. These findings suggest that targeting GSDMD may be beneficial in preventing and treating BPD and ROP in premature infants.

## Methods

### Materials

The following antibodies were used for immunostaining: anti-AIF1 (1:500 dilution) from ThermoFisher (Waltham, MA), anti-GSDMD (1:500 dilution) from Santa Cruz (Dallas, TX), anti-MAC-3 (1:20 dilution) from BD Biosciences (San Jose, CA), anti-vWF (1:50 dilution) from Dako (Carpinteria, CA), anti-α-SMA (1:100 dilution) from Sigma (Saint Louis, MO), anti-Ki67 (1:100 dilution), anti-neutrophil elastase (1:4000 dilution) and anti-RBPMS (1:500 dilution) from Abcam (Cambridge, MA). AlexaFluor-594 labeled isolectin IB4, TUNEL assay, and all qRT-PCR primers were purchased from ThermoFisher.

### Animals and study approval

The Animal Care and Use Committee of the University of Miami Miller School of Medicine approved the experimental protocol. All animals were cared for according to the National Institutes of Health guidelines for the use and care of animals. The study is reported in accordance with ARRIVE. GSDMD-KO mice (C57/B6)^13^ were obtained from Jackson Laboratory (Bar Harbor, ME). Heterozygote female and male mice were mated to produce newborn mice. Tail biopsy was done on newborn mice at postnatal day 7 for DNA extraction and PCR with primers to identify WT mice and homozygous KO mice which carry CRISPR/Cas9-derived knockout alleles that incorporates a 38 bp deletion in exon 5 of the GSDMD gene. Experiments were done with homozygous KO mice and their WT littermates.

### Hyperoxia-induced BPD model

Newborn GSDMD-KO mice and their WT littermates were exposed to RA (21% O_2_) or hyperoxia (85% O_2_) from P1 to P14 as previously described^15^. On P15, the pups were anesthetized by 0.1% isoflurane and their lungs were collected.

### Lung tissue section

Lungs were infused with 4% paraformaldehyde via a tracheal catheter at 20 cmH_2_O pressure for 5 min, fixed overnight, embedded in paraffin wax, and then sectioned.

### Assessment of GSDMD expression in lung tissues

Lung tissue sections were immunostained with an anti-GSDMD antibody to determine GSDMD protein expression.

### Assessment of lung inflammation

Macrophage infiltration was determined by immunostaining with an anti-MAC3 antibody, and neutrophil infiltration was assessed by immunostaining with an antibody for neutrophil elastase. The number of MAC-3-stained cells and neutrophil elastase-stained cells in the alveolar airspaces of lung tissue sections were counted from 5 random high power fields (HPF) taken from the 20 × objective on each slide^15^.

### Lung histology and morphometry

Lung tissue sections were stained by standard hematoxylin and eosin (H&E) method for histology and morphometry. The lung morphometric analysis was performed by a staff unaware of the experimental condition. For MLI assessment, five random images were taken with the 20 × objective on each HE-stained lung tissue section. The images were viewed under a field of equally spaced horizontal lines, and the MLI was calculated as the average of the total length of lines divided by the total intercepts of alveolar septa from each lung. For RAC measurement, five random terminal respiratory bronchioles were identified under the 20 × objective on each HE-stained lung tissue section. The number of distal air sacs transected by a line drawn from a terminal respiratory bronchiole to the nearest pleural surface was counted, and the RAC was calculated as the average number of distal air sacs from each lung section.

### Measurement of pulmonary vascularization and vascular remodeling

Double immunofluorescent staining for vWF, an endothelial marker, and α-SMA, a vascular smooth cell marker, was performed. Pulmonary vascular density was quantified by the number of vWF positive vessels (< 50 μm in diameter) per HPF in 5 randomly selected, non-overlapping, parenchymal fields on lung sections from each animal. Pulmonary vascular remodeling was assessed by the extent of muscularization of the peripheral pulmonary vessels. Muscularized vessels were defined by the presence of smooth muscle cells positively stained with α-SMA antibody in 50% or more of the vessel circumference. Five random, non-overlapping images on each lung section were viewed, and the percentage of muscularized peripheral pulmonary vessels was determined.

### Assessment of lung cell proliferation and death

Cell proliferation was assessed by immunofluorescent staining for Ki67, and the proliferating index was calculated as the average percentage of Ki67-positive nuclei in total nuclei in 5 random HPF on lung sections from each animal. Cell death was studied using a TUNEL assay and the cell death index was calculated as the average percentage of TUNEL-positive nuclei in total nuclei in 5 random HPF on lung sections from each animal^15,65^.

### Hyperoxia-induced ROP model

WT and GSDMD-KO newborn mice were exposed to RA from P1 to P17. To generate the ROP model, subsets of WT and GSDMD-KO mice were exposed to 75% oxygen from P7 to P12 and then placed on RA recovery^18,19^. Mice were anesthetized as described above, and the eyes and retinas were collected on P18.

### Assessment of GSDMD expression in retinas

Double fluorescent immunostaining for GSDMD and RBPMS was performed on retinal sections to colocalize GSDMD and RBPMS expression.

### Assessment of vasoobliteration and neonvascularization of retina

Whole-mount retinas were stained for endothelial cells with AlexaFluor-594 labeled isolectin IB4 and the degree of vasoobliteration and neovascularization was assessed as previously described^18^. Briefly, using Carl Zeiss ZEN Software, total avascular and total retinal areas were measured. Total avascular area in proportion to the total retinal area was used to calculate the total area of vasoobliteration in 6 retinas per group. Total neovascular areas were measured by the intensity of staining, and total neovascular area in proportion to the total retinal area was used to calculate the total area of neovascularization in 6 retinas per group.

### Assessment of retinal thickness

Eye sections were prepared, stained by H&E, and photographed under Zeiss ZEN Software. The thickness of each retinal layer was measured from three sections of each eye of 9–15 eyes/group using ImageJ software.

### Microglial assessment

Immunofluorescence with an antibody for AIF, a marker for microglia, was performed to detect active microglia and staining with AlexaFluor-594 labeled isolectin IB4 was performed to detect vasculatures on retinas.

### RNA isolation and real-time qRNA-PCR

Total RNA was extracted from frozen lung tissues and retinal tissues using the RNeasy Universal Mini Kit (Qiagen, Valencia, CA) according to the manufacturer’s instructions. RNA quality and integrity were verified using the Agilent 2100 Bioanalyzer (Agilent Technologies, Santa Clara, CA). All samples had RNA integrity numbers > 7. The Real-time qRT-PCR was performed on an ABI Fast 7500 System (Applied Biosystems, Foster City, CA) as previously described^65^. The expression levels of target genes were normalized to 18S rRNA.

### RNA sequencing

RNA-seq was performed by BGI Genomics (Hong Kong). A detailed description of the procedures was provided in the Supplemental Methods.

### Data management and statistical analysis

Data were expressed as mean ± SD and comparisons between groups were performed using one-way ANOVA and Tukey’s multiple comparison analysis. A *P*-value of 0.05 was considered significant.

## Supplementary Information

Supplementary Information.

## Data Availability

The datasets generated during the current study are available in GEO@ncbi.nlm.nih.gov under record GSE206087.
