# Supplementary material for: GSDMD deficiency ameliorates hyperoxia-induced BPD and ROP in neonatal mice
Source: Sci Rep. 2023 Jan 4;13:143. doi: 10.1038/s41598-022-27201-y (PMC9813139; doi:10.1038/s41598-022-27201-y)
Supplement: Supplementary file 1 — Supplementary Information. [file 41598_2022_27201_MOESM1_ESM.pdf]

ONLINE SUPPLEMENTS

Supplemental Methods

RNA Isolation and Sequencing: Total RNA was extracted from frozen lung and retinal tissues using the RNeasy Universal Mini Kit (Qiagen, Valencia, CA) according to the manufacturer's instructions. RNA quality and integrity were verified using the Agilent 2100 Bioanalyzer (Agilent Technologies, Santa Clara, CA). All samples had RNA integrity number > 7. RNA sequencing was performed by BGI Genomics (Hong Kong) with a read depth of 30 million reads per sample for 150 bp paired-end reads. The raw sequence read in FASTQ format were aligned to the mouse (*Mus musculus*) genome build mm_GRCm39_104 using kallisto (1) followed by gene summarization with tximport (2). After checking data quality, differential expression analyses comparing treatment groups to control and to each other were performed using DESeq2 with false discovery adjustment (3). Genes were considered differentially expressed based on their fold-change relative to control (= or >2), p-value (<0.05), and q-value (<0.1). Lists of differentially expressed genes were used for functional enrichment analysis of Gene Ontology and pathway terms using the clusterProfiler package in R (4, 5). Only unique terms associated with either induced or suppressed genes and at least 2 genes were reported. Upset plots of differentially expressed genes across different experimental conditions were generated using the UpsetR package (6).

1. Bray NL, et al. Near-optimal probabilistic RNA-seq quantification. *Nat Biotechnol*. 2016;34:525-527.
2. Soneson C, et al. Differential analyses for RNA-seq: transcript-level estimates improve

gene-level inferences. *F1000Res*. 2015;4:1521.

3. Love MI, et al. Moderated estimation of fold change and dispersion for RNA-seq data with DESeq2. *Genome Biol*. 2014;15(12):550.
4. Wu T, et al. ClusterProfiler 4.0: A universal enrichment tool for interpreting omics data. *Innovation (N Y)*. 2021;2:100141.
5. Yu G, Wang LG, Han Y, He QY. ClusterProfiler: an R package for comparing biological themes among gene clusters. *OMICS*. 2012;16(5):284-7.
6. Gehlenborg N. UpSetR: A more scalable alternative to venn and euler diagrams for visualizing intersecting sets. http://github.com/hms-dbmi/UpSetR.

Supplemental Figures

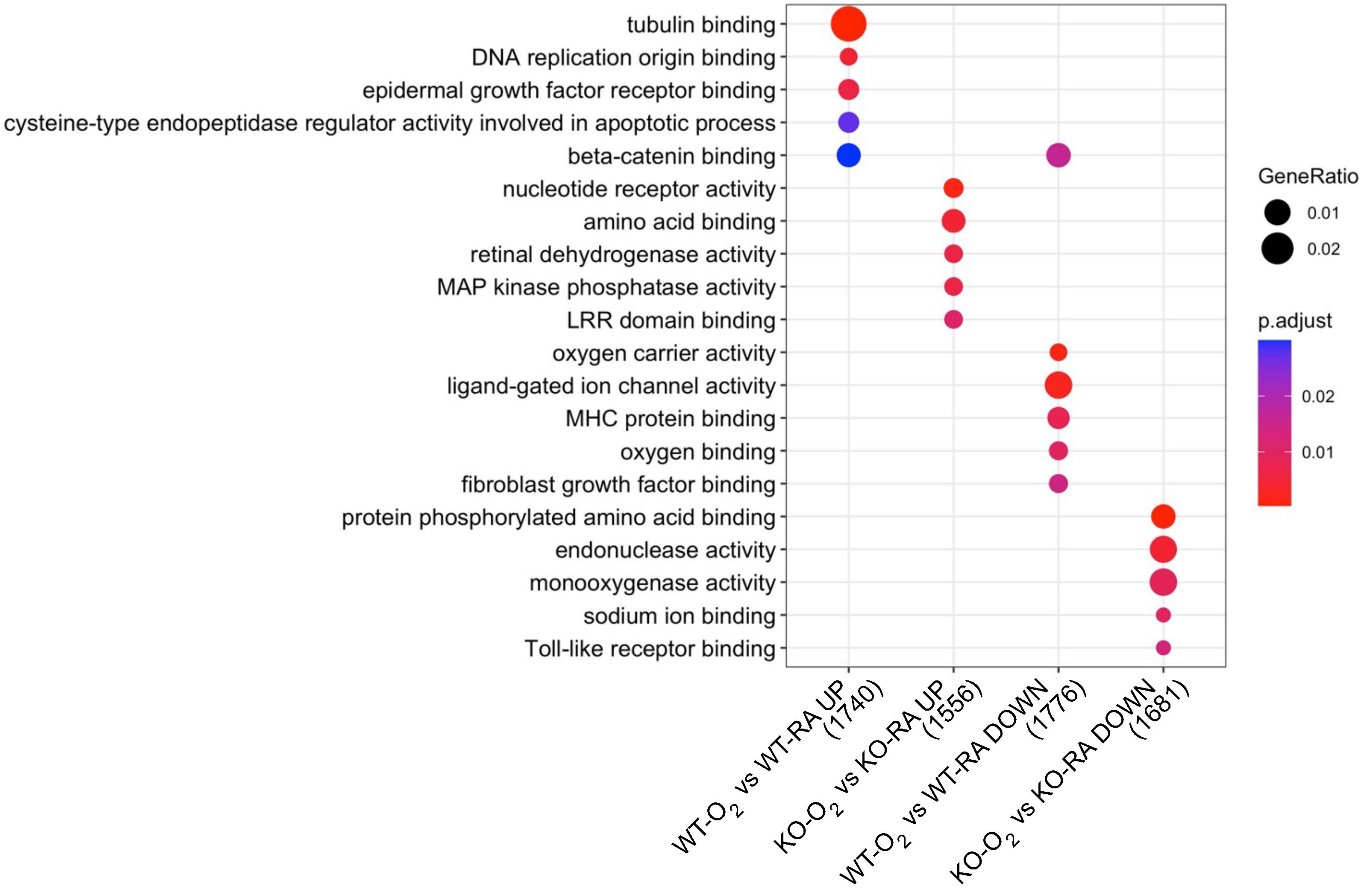

Supplemental Figure 1. Overrepresentation analysis and comparison of biological themes on ClusterProfile showing unique biological terms regulated by hyperoxia in WT and GSDMD-KO lungs. n = 3 animals/group.

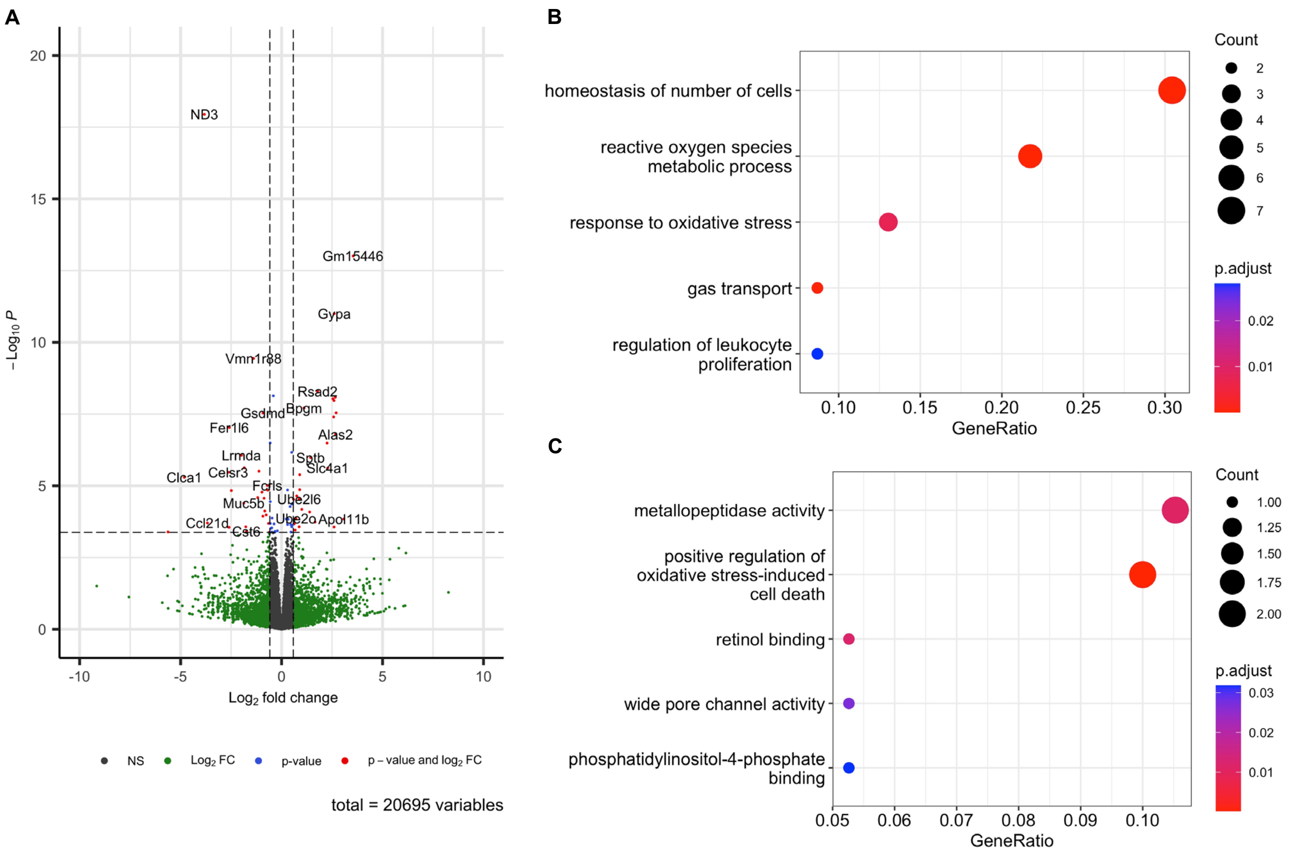

Supplemental Figure 2. A. Volcano plot of genes differentially expressed in lungs of hyperoxia-exposed GSDMD-KO mice compared to hyperoxia-exposed WT lungs. **B.** Gene set enrichment analysis of genes induced in hyperoxia-exposed GSDMD-KO mice. **C.** Gene set enrichment analysis of genes suppressed in hyperoxia-exposed GSDMD-KO mice. n = 3 animals/group.

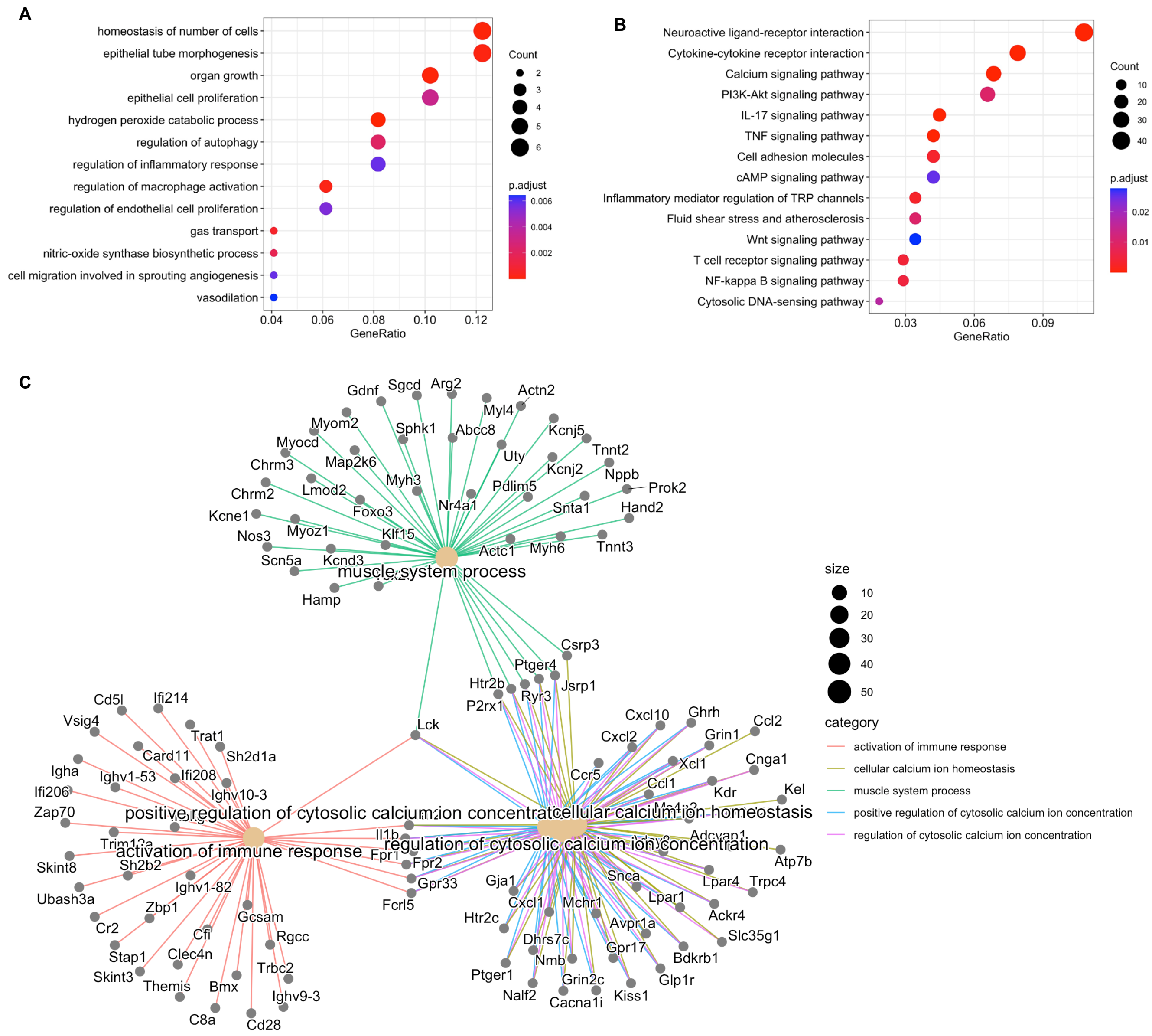

Supplemental Figure 3. A. Overrepresentation analysis for Gene Ontology term of genes identified to be modulated by GSDMD-KO in the lungs under hyperoxia exposure. **B.** Overrepresentation analysis for KEGG pathways of genes identified to be modulated by GSDMD-KO in the lungs exposed to hyperoxia. **C.** Network plot of the 5 top biological processes enriched for among genes regulated by GSDMD-KO in hyperoxia-exposed lungs. n = 3 animals/group.

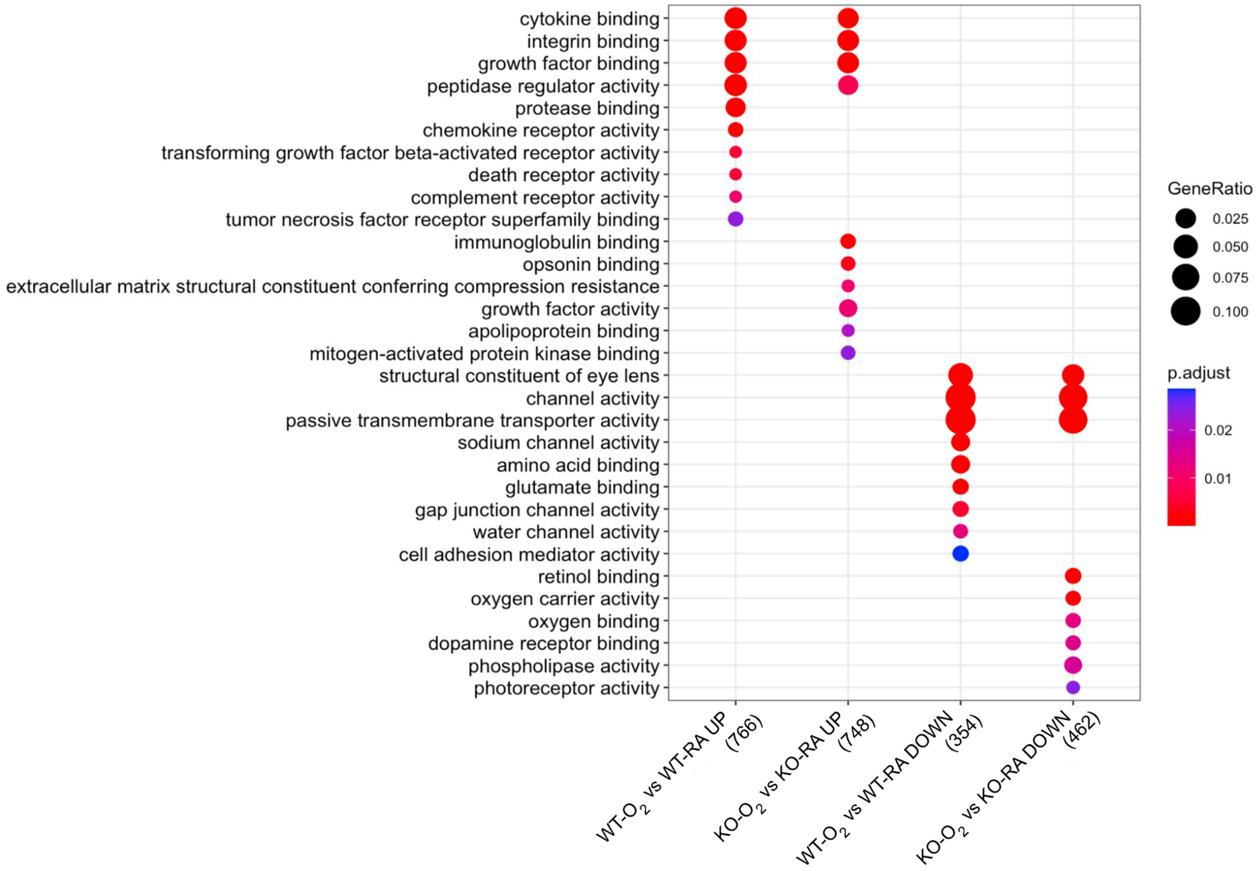

Supplemental Figure 4. Overrepresentation analysis and comparison of biological themes on ClusterProfile showing unique biological terms regulated by hyperoxia in WT and GSDMD-KO retinas. n = 3 animals/group.

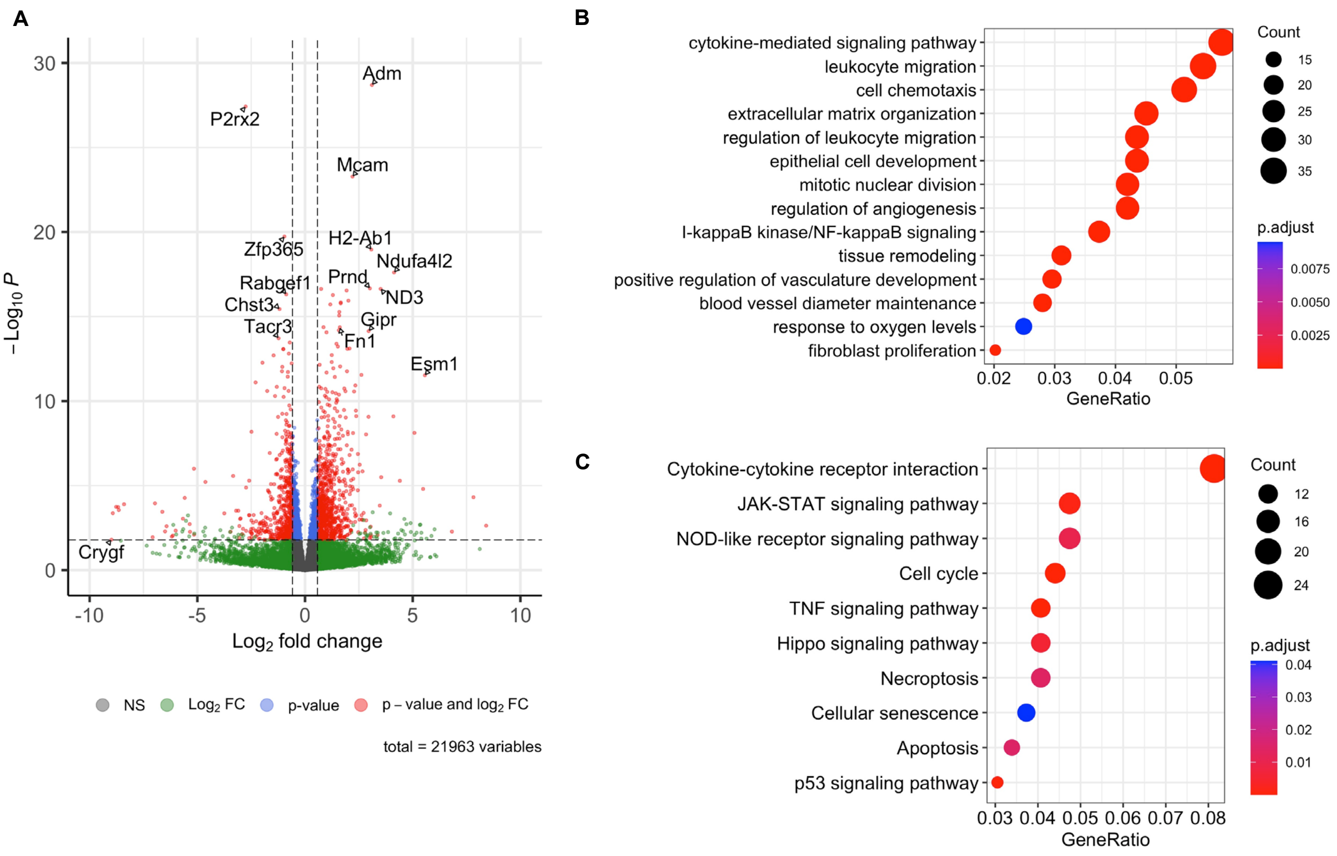

Supplemental Figure 5. A. Volcano plot of genes differentially expressed in the retinas of hyperoxia-exposed GSDMD-KO mice compared to hyperoxia-exposed WT mice. **B.** Gene set enrichment analysis of genes induced in hyperoxia-exposed GSDMD-KO retinas. **C.** Gene set enrichment analysis of genes suppressed in hyperoxia-exposed GSDMD-KO retinas. n = 3 animals/group.

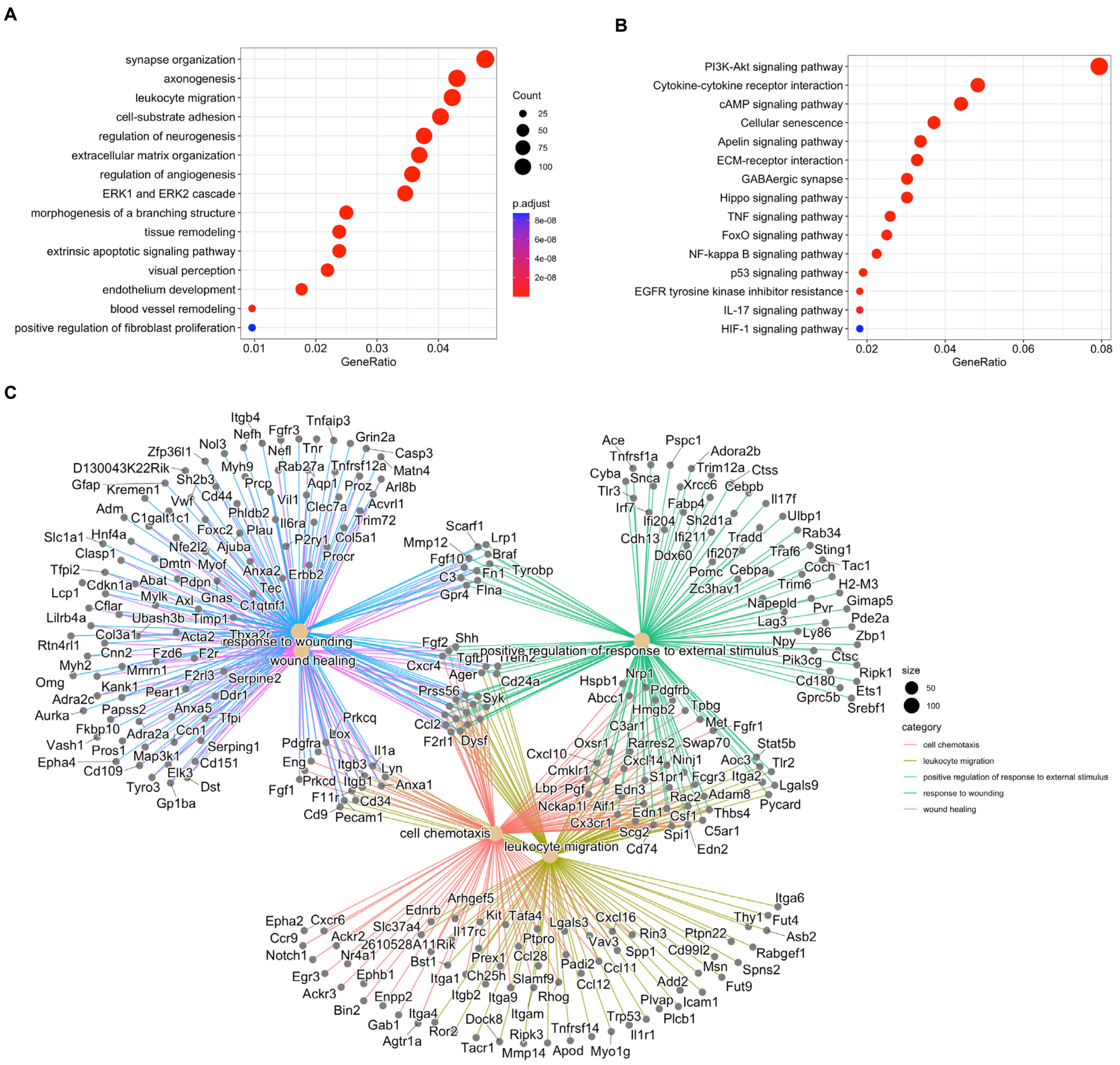

Supplemental Figure 6. A. Overrepresentation analysis for Gene Ontology term of genes identified to be modulated by GSDMD-KO in the setting of hyperoxia in the retinas. **B.** Overrepresentation analysis for KEGG pathways of genes identified to be modulated by GSDMD-KO in the setting of hyperoxia in the retinas. **C.** Network plot of the top 5 biological processes enriched for among genes regulated by GSDMD-KO in hyperoxia-exposed retinas. n = 3 animals/group.
